# Discovery of novel ancestry specific genes for androgens and hypogonadism in Million Veteran Program Men

**DOI:** 10.1038/s41467-025-57372-x

**Published:** 2025-05-02

**Authors:** Meghana S. Pagadala, Craig C. Teerlink, Guneet K. Jasuja, Madhuri Palnati, Tori Anglin-Foote, Nai-Chung N. Chang, Rishi Deka, Kyung M. Lee, Fatai Y. Agiri, Tiffany Amariuta, Tyler M. Seibert, Brent S. Rose, Kathryn M. Pridgen, Julie A. Lynch, Hannah K. Carter, Matthew S. Panizzon, Richard L. Hauger

**Affiliations:** 1 Research Service, VA San Diego Healthcare System, San Diego, CA USA; 2 Medical Scientist Training Program, University of California San Diego, La Jolla, CA USA; 3 Biomedical Science Program, University of California San Diego, La Jolla, CA USA; 4 VA Informatics and Computing Infrastructure (VINCI), VA Salt Lake City Healthcare System, Salt Lake City, UT US; 5 Department of Internal Medicine, Division of Epidemiology, University of Utah School of Medicine, Salt Lake City, UT US; 6 Center for Healthcare Organization and Implementation Research (CHOIR), VA Bedford Healthcare System, Bedford, MA US; 7 Section of General Internal Medicine, Boston University School of Medicine, Boston, MA US; 8 Department of Health Law, Policy, and Management, Boston University School of Public Health, Boston, MA US; 9 Department of Radiation Medicine and Applied Sciences, University of California San Diego, La Jolla, CA USA; 10 Department of Psychiatry, University of California San Diego, La Jolla, CA USA; 11 Department of Medicine, University of California San Diego, La Jolla, CA USA; 12 Halicioglu Data Science Institute, University of California San Diego, La Jolla, CA USA; 13 Department of Radiology, University of California San Diego, La Jolla, CA USA; 14 Department of Bioengineering, University of California San Diego, La Jolla, CA USA; 15 Department of Urology, University of California San Diego, La Jolla, CA USA; 16 Center for Behavior Genetics of Aging, University of California San Diego, La Jolla, CA USA; 17 Center of Excellence for Stress and Mental Health (CESAMH), VA San Diego Healthcare System, San Diego, CA USA

**Keywords:** Genome-wide association studies, Gene expression profiling, Endocrine system and metabolic diseases

## Abstract

Given the various roles of testosterone in men’s health, we conducted a multi-ancestral genetic analysis of total testosterone, free testosterone, SHBG, and hypogonadism in men within the Million Veteran Program (MVP). Here we identified 157 significant testosterone genetic variants, of which 8 have significant ancestry-specific associations. These variants implicate several genes, including *SERPINF2*, *PRPF8*, *BAIAP2L1*, *SHBG*, *PRMT6*, and *PPIF*, related to liver function. Genetic regulators of testosterone have cell type-specific effects in the testes, liver, and adrenal gland and are associated with disease risk. We conducted a meta-analysis amongst ancestry groups to identify 188 variants significantly associated with testosterone, of which 22 are novel associations. We constructed genetic scores for total testosterone, SHBG levels, and hypogonadism and find that men with higher testosterone genetic scores have lower odds of diabetes, hyperlipidemia, gout, and cardiac disorders. These findings provide insight into androgen regulation and identify novel variants for disease risk stratification.

## Abstract (web summary)

Here, the authors perform a genome-wide association study to identify 157 significant testosterone genetic variants in male veterans, of which 8 are ancestry-specific. Men with higher testosterone genetic scores have lower odds of diabetes, hyperlipidemia, gout, and cardiac disorders.

## Introduction

Testosterone is an anabolic steroid synthesized by the testes which acts as the primary sex hormone in men^1–3^. Classical, well-established roles of testosterone include regulating sexual development during puberty, spermatogenesis, erythropoiesis, muscular strength, and bone density mass^4,5^; however, testicular androgens also have extensive physiological effects on cardiovascular, metabolic, hepatic, immune, and brain function^2,6–8^. These effects are primarily mediated by the androgen receptor (AR), which is encoded on the X chromosome and is widely expressed in many tissues throughout the body, particularly in the testes, prostate, liver, adipose tissue, skeletal muscle, heart, kidney, and brain^2,9^. Progressive reduction in testicular androgen steroidogenesis begins in the third to fourth decade of a man’s life, with a roughly 1–3% decrease in circulating levels of total testosterone per year^1,2,10,11^. The development of age-related testosterone deficiency or hypogonadism contributes to sexual dysfunction, frailty, sarcopenia, fatty liver disease, cardiovascular disorders, chronic kidney disease, obesity, and metabolic dysregulation^1,2,10–19^. Testosterone deficiency has also been linked to depression, neurodegeneration, cognitive impairment, and Alzheimer’s disease risk during male aging^20–25^.

Given the various roles of testosterone in men’s health, ascertaining genetic determinants of testosterone is vital. Approximately 50–60% of the testosterone in circulation is bound to sex hormone-binding globulin (SHBG), and 40–50% is bound to albumin^10^. Unbound testosterone (1–2%), which is termed free testosterone, and SHBG also function as important modulators of androgen action. To date, genome-wide association studies (GWAS) of testosterone, SHBG, and low testosterone levels have unveiled associations with variants in or near genes such as *SHBG, JMJD1C, FKBP4, REEP3*, and *FAM9B* and suggest that the SNP-based heritability for total and free testosterone is ~ 20% and ~ 15%, respectively^26–34^. These GWAS studies have been conducted primarily in European or Asian ancestry groups, despite the differences in testosterone levels that have been observed in men of different ancestries^35–37^. Few studies have analyzed ancestry-specific and trans-ancestry genes regulating total testosterone, free testosterone, SHBG, and hypogonadism in a diverse male cohort.

In this study, we present a GWAS of total and free testosterone levels, SHBG levels, and hypogonadism in the Million Veteran Program (MVP), a large, multi-ethnic genetic biorepository from a hospital-based population. As a hospital-based population, MVP has a higher disease prevalence of testicular dysfunction/hypofunction, type 2 diabetes, obesity, hyperlipidemia, prostate cancer, and cardiovascular diseases compared to other population studies, thus offering unique insights into the interaction of diseases and testosterone-regulating genes in a clinical setting. We identify associations with total testosterone, free testosterone, SHBG levels, and hypogonadism risk within European (EUR), African (AFR), admixed American (AMR), and East Asian (EAS) ancestry groups. In colocalization analyses, we find that several variants associated with testosterone levels and hypogonadism risk are located in or near genes related to liver function (*UGT2B17, BRI3, PRMT6, PPIF)*. Lastly, we construct and validate genetic scores and genetic risk scores (GS/GRSs) to estimate testosterone, SHBG levels, and hypogonadism and find significant associations with metabolic conditions, such as hyperlipidemia, gout, and diabetes, as well as cardiac and liver disorders.

## Results

### Curation of Laboratory Measures and Identification of Hypogonadism

We identified male participants within the MVP with documented total testosterone levels (*n* = 265,038), free testosterone levels (*n* = 110,627), and SHBG levels (*n* = 43,325). After initial data curation and preprocessing, morning total testosterone, free testosterone, and SHBG levels were available for 139,226, 49,709, and 19,635 men, respectively (Supplementary Fig. S1 and Table 1). MVP phenotyping data is dependent on endocrine lab tests being ordered in a clinical setting, resulting in different sample sizes for each hormone level. Average total testosterone (avg +/− std dev: 389 [196–582] ng/dL), free testosterone (9.4 [− 26–45] ng/dL), and SHBG (42 [15–68] nmol/L) levels were within the physiologically normal range for men (Supplementary Fig. S2A–C). Notably, our analysis confirmed the well-documented trend of decreasing total and free testosterone levels and increasing SHBG levels with age (Supplementary Fig.  S2D–F)^38,39^.

**Table 1:** Sample Sizes for GWAS Analysis of Testosterone Levels and Hypogonadism

Ancestry Groups	Genotype	Total	Free	SHBG	Hypogonadism
All	588177	139226	49709	19635	146720
European	421613	95184	33470	13893	102635
African	105305	27521	9863	2960	27736
Admixed American	50829	13443	5256	2081	13328
East Asian	9154	1836	545	471	1690

Table of sample sizes for all EUR, AFR, AMR, and EAS MVP men used for GWAS of total testosterone (total), free testosterone (free), SHBG, and diagnosis of hypogonadism. Also, included number of individuals for which genotypes were available.

MVP ancestry groups were assigned using a recently published random-forest clustering with the 1000 Genomes Project and the Human Genome Diversity Project (1kGP + HGDP) reference panels^40^. Differences in total testosterone, free testosterone, and SHBG levels based on ancestry were small (Supplementary Fig. S2G–I). Significantly higher total testosterone levels were observed within the AFR ancestry group (406.6 [200.5–612.7] ng/dL) compared to EUR (382.9 [193.5–572.4] ng/dL), EAS (382.8 [201.8–563.7] ng/dL), and AMR (395.1 [201.9–588.4] ng/dL) ancestry groups (*p* < 0.001) (Supplementary Fig. S2G). In addition, the EAS ancestry group had lower SHBG levels (30.8 [12.4–49.3] nmol/L) compared to other ancestry groups (EUR: 42.7 [16.9–68.6] nmol/L, AFR: 41.2 [11.3–71.2] nmol/L, AMR: 36.4 [11.9–60.9] nmol/L; *p* < 0.001) (Supplementary Fig. S2I).

We extended our analysis to hypogonadism, a condition characterized by abnormally low testosterone levels. Based on our criteria for hypogonadism diagnosis (Methods), ~ 25% of the study population have hypogonadism; the incidence was higher in the AFR and AMR ancestry groups and lower in the EAS ancestry group (Supplementary Fig. S2J). We confirmed that men with hypogonadism had lower total testosterone levels compared to matched controls (Supplementary Fig. S2K).

Before conducting GWAS, we estimated heritability or the proportion of phenotypic variance due to genetic variance for testosterone phenotypes using linkage disequilibrium score regression (LDSC) analysis^41^. Total testosterone, SHBG, and hypogonadism heritability estimates were 9–12%, 20–50%, and 5–9%, respectively (Supplementary Table S1). The positive genetic correlation was observed for total testosterone with free testosterone and SHBG levels in the EUR ancestry groups, while negative genetic correlations were observed between total testosterone and hypogonadism in the EUR, AFR, and AMR ancestry groups (Supplementary Table S2).

### Genetic associations of laboratory measures and hypogonadism

We conducted GWAS with total testosterone levels, free testosterone levels, SHBG levels, and hypogonadism within MVP ancestry groups^40^. We identified men of EUR (95,184), AFR (27,521), AMR (13,443), and EAS (1816) ancestry for total testosterone analysis. Cohort sizes for free testosterone, SHBG, and hypogonadism are given in Table 1.

We discovered 157 significant genetic associations for total and free testosterone, SHBG, and hypogonadism (Supplementary Fig. S3 and Supplementary Data 1). Of these, the most significant associations were from analyses of EUR (98) and AFR (35) ancestry groups; however, significant associations in AMR (16) and EAS (8) groups were also identified. Eight variants had significantly different effect size estimates when comparing ancestry groups (“Methods”); most variants had different effect sizes in the EAS (5) ancestry group compared to EUR (0), AFR (1), and AMR (2) ancestry groups (Supplementary Fig. S4). Generally, however, variants had similar effects on hormone levels across ancestry groups.

We performed a SuSiE fine-mapping analysis of 157 associations to determine the proportion of variants with a high probability of being causal. An important advantage of SuSiE is that this analysis method allows for multiple causal variants in a region, in contrast to other causality inference methods^42^. The associations of 49 (31.2%) lead variants had a high PIP > 0.10. Several of these variants were missense variants, implicating genes, such as *SERPINA1, NR2F6, FKBP4, SLCO1B1, HGFAC*, and *UGT2B15*.

To replicate our results, we compared significant associations from MVP EUR GWAS with total testosterone, free testosterone, and SHBG associations from the UK Biobank analyses performed by Ruth et al. with total testosterone, free testosterone, and SHBG (hypogonadism GWAS was not conducted)^26^. Of the MVP EUR GWAS variants, 87.2% (89.1% total testosterone, 100% SHBG, 33.3% free testosterone) were replicated in the UK Biobank. Six MVP EUR variants were not replicated, but effect sizes and minor allele frequencies were generally consistent (Supplementary Data 2 and Supplementary Fig. S5). We also compared MVP AFR GWAS with associations with total testosterone, free testosterone, and SHBG levels for 1,257 African-American men in the Multi-Ethnic Study of Atherosclerosis (MESA) cohort. Only two variants (14%) were nominally significant (*p* < 0.05) in the MESA cohort, likely due to its smaller sample size. However, effect size estimates of associations were consistent with MVP associations (Pearson *r* = 0.64, *p* < 0.005) (Supplementary Fig. S6).

### Gene expression in specific cell types and disease risk

**Fig. 1:**
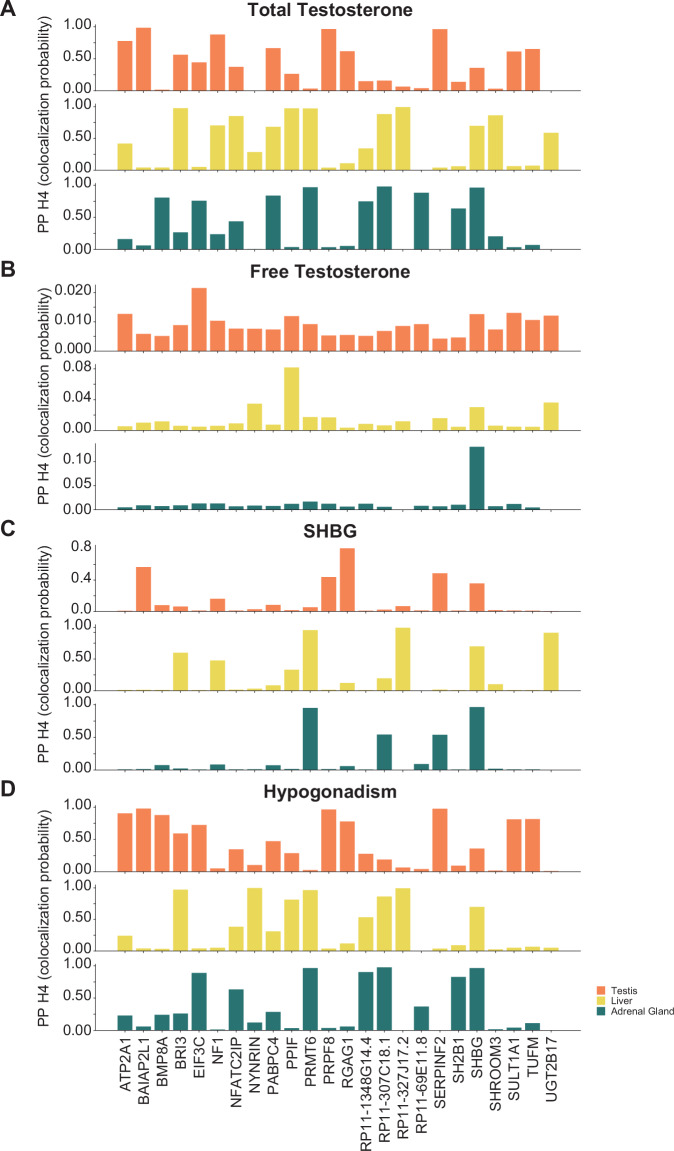
Testosterone variants affect gene expression in specific cell types. Bar plot of colocalization probability (PP.H4) with GTEx gene expression in testes (orange), liver (yellow), adrenal gland (green) for (**A**) total testosterone, (**B**) free testosterone, (**C**) sex-hormone binding globulin (SHBG) levels and (**D**) hypogonadism. Full colocalization results are given in Supplementary Data 3.

To identify candidate genes influencing testosterone levels, we performed colocalization analysis using GTEx cis-eQTL gene expression summary statistics for androgen-regulated tissues and/or sites and MVP EUR GWAS. The colocalization analysis estimates the probability of a shared causal signal between *cis-*eQTL and GWAS analyses of total testosterone levels, free testosterone levels, SHBG levels, and hypogonadism^43^. In total, we discovered 24 genes with high probability (PP.H4 > 0.8) of shared causal variants between their expression and total testosterone levels (15), hypogonadism (17) and SHBG levels (5) (Fig. 1). Genes such as *SHBG, SERPINF2, PRPF8, PRMT6, BRI3, BAIAP2L1, NF1, BMP8A, PABPC4, TUFM, PPIF, SULT1A1, NYNRIN* and *RP11-327J17.2* have significant shared colocalization signal with total testosterone levels, SHBG levels, or hypogonadism (Supplementary Data 3). *NYNRIN*, *TUFM*, and *SH2B1* were uniquely identified by colocalization analysis of hypogonadism, while *NF1, PABPC4*, and *SHROOM3* were uniquely identified through colocalization analysis of total testosterone. We found that some gene associations (e.g., *SULT1A1, PRPF8, and SERPINF2)* were specific to the testes while *BRI3* and *NYNRIN* had liver cell type-specific effects. Although the testes are the primary source of testosterone production, these results identify the potential involvement of other tissues and organs for testosterone regulation.

To evaluate the disease relevance of the testosterone and hypogonadism variants, we performed PheWAS analyses with 1875 phecodes in the MVP. Testosterone variants were associated with diabetes (*n* = 8), hyperlipidemia (*n* = 8), gout (*n* = 6), and liver disease (*n* = 7). To determine whether PheWAS associations were due to testosterone changes, we ran colocalization analyses to determine the probability of shared causal variants between testosterone and disease. Approximately 85.9% (367/427) of the PheWAS associations had a greater than 80% probability of shared causal variants (Supplementary Data 4).

### MVP Meta-analysis of laboratory measures and hypogonadism

**Fig. 2:**
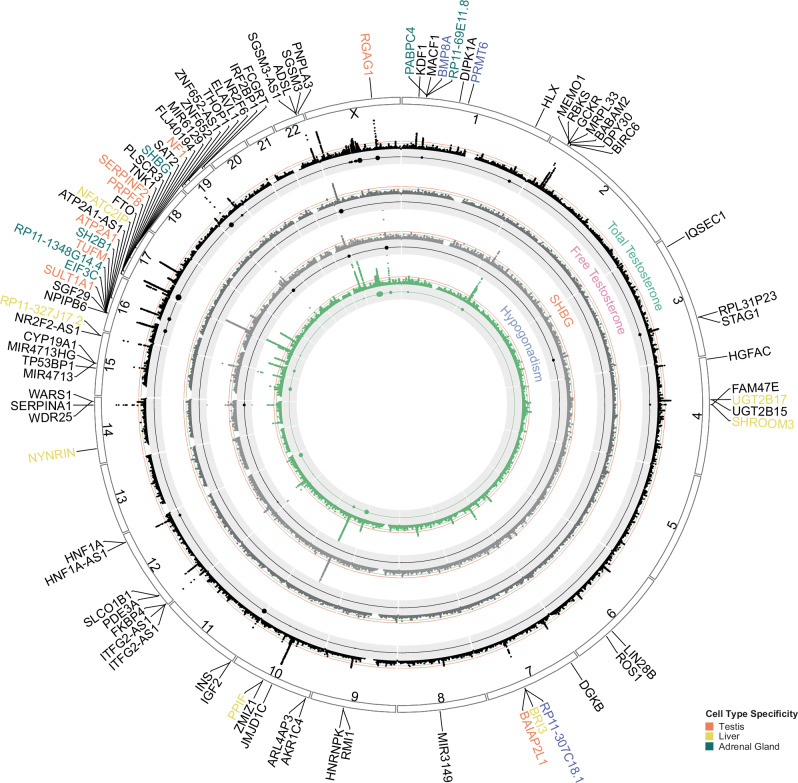
GWAS of Testosterone levels and hypogonadism in MVP men. Circos plot of METAL *p*-values (-log base 10) of GWAS for total testosterone, free testosterone, SHBG, and hypogonadism in the MVP. The green line indicates a suggestive threshold (*p* < 10^−6^), and the red line indicates a genome-wide significance threshold (*p* < 5 × 10^−^^8^). The gray band correspond to Cochran's Q statistic to measure heterogeneity. Genes implicated through colocalization analysis (PP H4 > 0.8) are labeled on the outer ring and colored by cell type localization signal: liver (yellow), adrenal gland (green), testes (orange), multiple cell types (purple). A complete list of associations and nearby genes can be found in Supplementary Data 6.

**Fig. 3:**
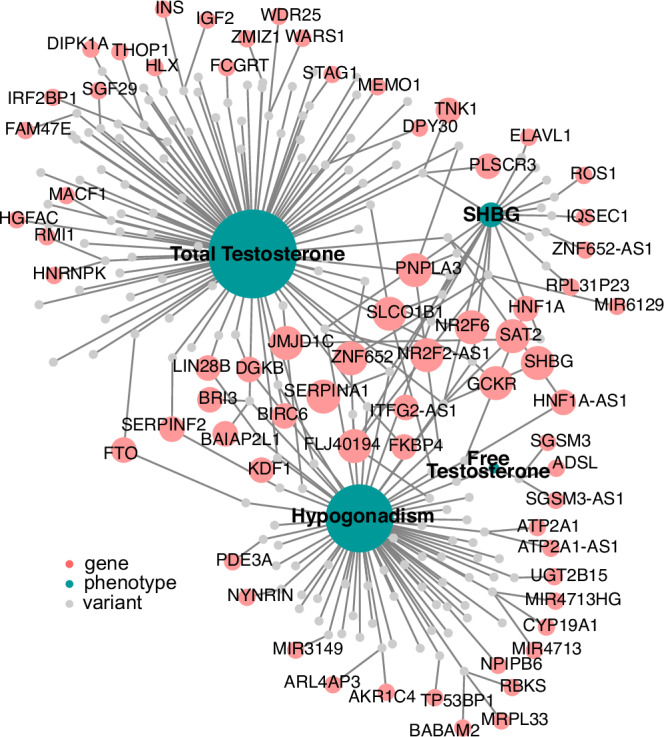
Genes implicated in total testosterone, free testosterone, SHBG and hypogonadism GWAS analysis. Implication performed using variant effect predictor (VEP). Network plot of genes (coral), variants (gray), and phenotypes (teal). Scatter points are scaled by the number of edges.

After the ancestry GWAS was completed, we conducted a meta-analysis to identify a consensus set of 188 associations affecting total testosterone, free testosterone, SHBG, and hypogonadism (Fig. 2 and Supplementary Data 5). Most variants were associated with total testosterone and hypogonadism, as these analyses had a larger sample size and thus were more powered to identify significant associations. Generally, effect sizes were consistent across ancestry groups (Supplementary Fig. S7). We identified several genes implicated in previous testosterone GWAS analyses, such as *JMJDC1, SERPINF2, BAIAP2L1, FKBP4*, and *SHBG* (Fig. 3)^26–34^. Most variants were non-coding; 14 associations were missense variants, resulting in protein changes. Several missense variants were in liver function genes, such as *SERPINA1*, *NR2F6, GCKR, SLCO1B1, PNPLA3, HGFAC* and *UGT2B15*. In fact, 31% (53/171) of lead variants were significantly associated with AST and ALT levels, underscoring the effects of variants on liver function.

We compared our variants to previous analyses of total testosterone, free testosterone, and SHBG levels conducted in the UK Biobank^44^. Notably, all participants in the UK Biobank have total testosterone, SHBG, and bioavailable testosterone measured upon enrollment, whereas phenotype measurements were only available in MVP if ordered by a physician. The MVP cohort has a higher disease prevalence of testicular dysfunction/hypofunction, type 2 diabetes, obesity, hyperlipidemia, prostate cancer, gout, and sleep apnea compared to the UK Biobank (Table 2). 64.1% (75/117) of variants were significant in UK Biobank analyses. Since a majority of variants were found on chromosome X, we removed X chromosome variants in LD (R^2^ > 0.2) with other X chromosome novel variants, leaving 22 novel MVP variants/loci (Supplementary Data 6).

**Table 2:** Differences in MVP and UK Biobank populations

	UK Biobank	MVP
age at enrollment (median [min-max])	58 [37–73]	64 [18–110]
% female/male	54.4/45.6	9.2/90.8
% diabetes	15.1%	46.6%
% hypertensive diseases	37.0%	69.5%
% lipid disorders	21.1%	71.3%
% testicular dysfunction	0.1%	2.9%
% renal failure	14.1%	24.5%
% liver disease	5.1%	19.7%

Hypogonadism summary statistics for the UK Biobank were not available. However, only 416 men had a diagnosis code of testicular hypofunction (International Classification of Disease [ICD-10] E29.1, 0.09%) in the UK Biobank in contrast to the 25% of men within the MVP cohort who meet this study’s definition of hypogonadism; thus, a case-control GWAS in UK Biobank would be severely underpowered.

### GS and GRS Construction and validation

**Fig. 4:**
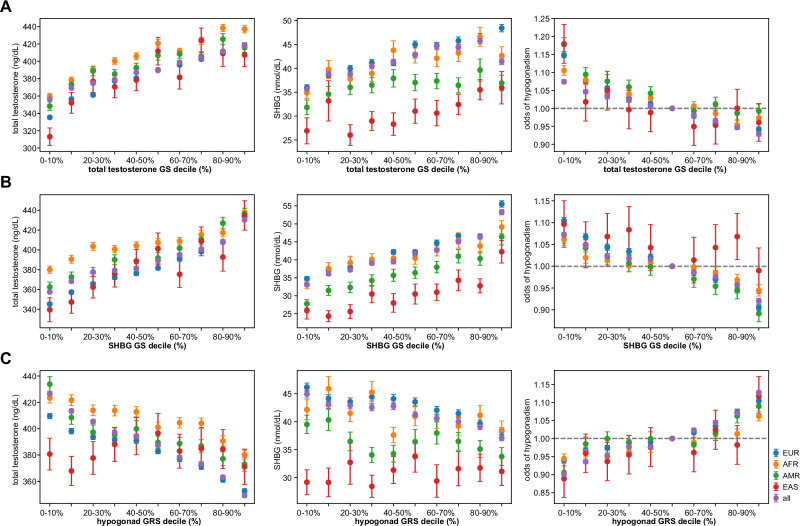
Total testosterone, SHBG GS and hypogonadism GRS construction and internal validation. Decile plots of total testosterone GS (**A**), SHBG GS (**B**) and hypogonadism GRS (**C**) and corresponding levels of total testosterone (ng/dL), SHBG levels (nmol/dl) and odds of hypogonadism, respectively, all MVP (*n* = 658582) and for EUR (*n* = 454996), AFR (*n* = 122068), AMR (*n* = 56513) and EAS (*n* = 10518) ancestry groups. Data are presented as mean values +/− standard error of the mean (SEM).

Given the number of disease associations with identified testosterone variants, we constructed GS/GRS including significant meta-analysis associations for each phenotype. The GRS was used to evaluate hypogonadism risk, while the GS evaluated lab values rather than risk. The GS effectively stratified MVP men by levels of total testosterone and SHBG levels (Fig. 4A, B). Free testosterone GS was not evaluated because < 5 significant variants were identified. The odds of hypogonadism were evaluated for each decile of hypogonadism GRS (Fig. 4C). The odds of hypogonadism in top deciles for European (EUR) (1.10 [1.10–1.11]), African (AFR) (1.06 [1.05– 1.08]) and American (AMR) (1.09 [1.07–1.11]) ancestry groups were significantly greater than those in bottom deciles for each ancestry group. We validated our total testosterone and SHBG GRS in the UK Biobank (Supplementary Fig. S8A, S8B). The hypogonadism GRS did not appear to stratify odds in the UK Biobank (Supplementary Fig. S8C).

Top significant associations from PheWAS analyses included metabolic (Phecode 250-Type 2 Diabetes, Phecode 272-Hyperlipidemia, Phecode 274-Gout, Phecode 278-Obesity), liver (Phecode 571, 572, 573) and cardiac disorders (Phecode 401, 411, 427). To determine if total testosterone and SHBG levels were associated with disease risk, we first conducted Mendelian randomization^45,46^ studies with MVP EUR results and published GWAS studies of gout, type 2 diabetes, obesity, metabolic dysfunction-associated steatotic liver disease (MASLD), hyperlipidemia, and chronic heart failure (Supplementary Data 7). Lower levels of total testosterone and SHBG were associated with a higher risk of hyperlipidemia, while hypogonadism was associated with a higher risk of hyperlipidemia. Hypogonadism was causally associated with a higher risk of gout (effect size = 0.40, *p* < 0.03), while higher levels of SHBG was associated with a lower risk of chronic heart failure (effect size = − 0.09, *p* < .04) (Supplementary Fig. S9).

**Fig. 5:**
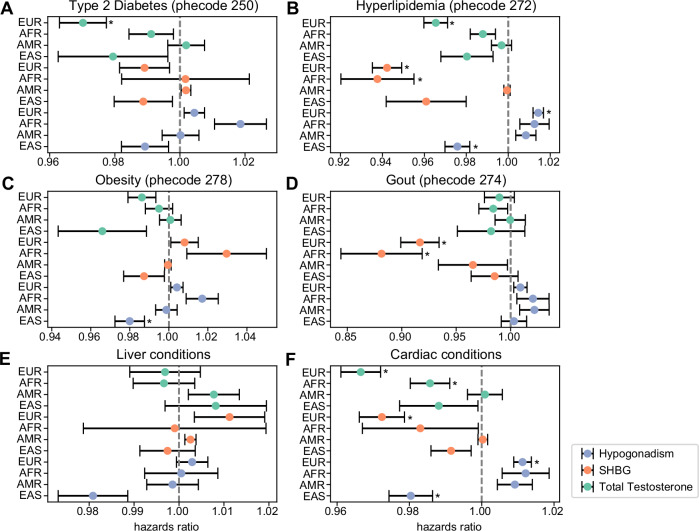
Association of Total Testosterone, SHBG GS and Hypogonadism GRS with Disease Risk in MVP. CoxPH Hazards ratio of association of total testosterone GS, SHBG GS and hypogonadism GRS with diabetes (**A**), hyperlipidemia (**B**), obesity (**C**), gout (**D**), liver disorders (**E**) and cardiac disorders (**F**) in EUR (total testosterone *n* = 292983, SHBG *n* = 373815, hypogonadism *n* = 313728), AFR (total testosterone *n* = 69927, SHBG *n* = 94358, hypogonadism *n* = 75851), AMR (total testosterone *n* = 34304, SHBG *n* = 45567, hypogonadism *n* = 37089) and EAS (total testosterone *n* = 6985, SHBG *n* = 8319, hypogonadism *n* = 7405) ancestries in the MVP, excluding individuals from discovery analyses. Data are presented as mean values +/− SEM. Asterisk (*) indicates associations significant after Benjamini-Hochberg multiple test correction. Full association statistics are given in Supplementary Data 8.

To determine whether our constructed and validated GS could reveal disease associations in MVP ancestry groups, we conducted Cox proportional hazards analyses with the total testosterone GS, the SHBG GS, and the hypogonadism GRS (Fig. 5 and Supplementary Data 8). Higher total testosterone levels were associated with lower risk of type 2 diabetes (0.97 [0.96-0.98], *p* < 10^-4^) and cardiac disorders (0.97 [0.96-0.97], *p* < 10^−9^) in the EUR ancestry group (Fig. 5A, F). Higher SHBG levels were associated with a lower risk of gout (0.92 [0.90–0.93], *p* < 10^-6^) in the EUR ancestry group (Fig. 5D). Risk of hyperlipidemia was lower in the EAS ancestry group with a higher risk of hypogonadism (0.98 [0.97-0.98], *p* < 10^-4^) while risk of hyperlipidemia was higher in the EUR ancestry group with higher risk of hypogonadism (1.01 [1.01-1.02], *p* < 10^-10^) (Fig. 5B). This differential risk may reflect baseline ancestry-specific difference in metabolic conditions; previous studies have noted differences in lipid levels in Asian individuals^47–50^ Given the number of liver genes linked with variants, we analyzed associations between GS/GRS and different liver disease categories (chronic liver disease, ascites, necrosis). No significant associations were found after multiple test corrections (Supplementary Fig. S10). As expected, higher total testosterone GS were associated with a significantly lower risk of testicular dysfunction, while higher hypogonadism GRS were associated with a higher risk of testicular dysfunction in all ancestry groups except the EAS ancestry group (Supplementary Fig. S11A). No significant associations were found between testosterone GS and prostate cancer or dementia (Supplementary Fig. S11B, 11C). These results highlight the role of testosterone and hypogonadism in many diseases.

## Discussion

Utilizing data from the MVP male cohort, we completed a large, multi-ancestral GWAS of total testosterone, free testosterone, SHBG levels, and hypogonadism. We had data for over 10,000 men for each phenotype and identified 157 associations from ancestry-specific GWAS and 188 from MVP meta-analysis. Comparing our findings with the large-scale UK Biobank study by Ruth et al., we found a 64.1% overlap amongst MVP variants associated with total testosterone, free testosterone, and SHBG (*p* < 4.3 × 10^−4^)^26^. After clumping X chromosome variants, the 22 novel variant regions we identified were mostly located on the X chromosome, an important chromosome for androgen receptor regulation. We also identified 9 ancestry-specific signals in men of EUR, AFR, AMR, and EAS ancestry located across many chromosomes (1, 2, 3, 4, 7, 10, 19, X). These results underscore the shared genetic regulation of testosterone and hypogonadism in men, but also unique ancestry-specific associations that may inform future health management and clinical treatment.

Protein-coding variants in several liver genes, such as *PNPLA3, GCKR, SLCO1B1, SERPINA1, HGFAC*, and *UGT2B15*, were significantly associated with total testosterone levels and hypogonadism risk. The *SERPINA1* gene encodes alpha-1 antitrypsin (ATA1), which is a serine protease inhibitor highly expressed in the liver; *NR2F6* encodes a DNA-binding transcription factor that upregulates fatty acid transport and uptake into hepatocytes. *SERPINA1* and *NR2F6* variants have been linked with ALT/AST elevation and MASLD susceptibility^51–55^. *GCKR* and *HGFAC* are also critical for liver glucose utilization and metabolism in the liver^56–58^.

Our colocalization analysis further supported a link between liver gene expression and testosterone. *NYNRIN*, a liver enzyme gene, was uniquely identified in colocalization analysis of hypogonadism and gene expression^59^. *UGT2B17*, another hepatic gene, was also identified in the colocalization of SHBG. *UGT2B17* encodes a uridine glucuronosyltransferase, which is involved in testosterone metabolism. In addition, we detected strong colocalization signals for *PRMT6* and *SHBG* in the liver and adrenal gland that were linked to total testosterone and hypogonadism. SHBG is the principal glycoprotein transporting testosterone and synthesized in the liver^10^. SHBG levels have been found to have an inverse relationship with MASLD^58^. PRMT proteins, including PRMT6, have a clinically significant role in hepatic fibrosis and cancer^60^. *PRMT6* encodes arginine methyltransferase that regulates androgen receptor translocation^61^. Androgen receptors are expressed in both hepatocytes and adrenocortical cells^9^. Our GWAS findings, which demonstrate the overlap between genes linked to testosterone levels and their expression in the liver and adrenal gland, suggest that genetically regulated testosterone may have an important role in normal liver and adrenal function, as well as in hepatic diseases, or that variation in liver and adrenal function can modify testosterone levels and hypogonadism risk.

Using the GS for total testosterone and SHBG and the hypogonadism genetic risk score (GRS) that we developed from our GWAS analyses, we identified associations between testosterone gene variants and the risk of certain diseases, especially metabolic diseases such as diabetes, gout, and hyperlipidemia. Low testosterone levels are associated with increased insulin resistance in men with both type 1 and type 2 diabetes, and insulin sensitivity and testosterone levels are hypothesized to influence one another^62^. The association between testosterone GS and diabetes risk identified in this study is consistent with the findings of Ruth et al., in which a Mendelian randomization analysis suggested that higher testosterone reduced the risk of type 2 diabetes in men^26^.

Our study did not find a significant link between higher total testosterone GS and dementia which was unexpected in light of research on the relationship of testosterone and cognitive decline during aging and previous findings that testosterone deficiency may be a risk factor for Alzheimer’s disease^21,63–66^. Underlying the memory-enhancing action of testosterone, preclinical research has found that testosterone-activated androgen receptor signaling in the hippocampus can induce synaptic plasticity, promote neuroprotection, and stimulate neurogenesis^67,68^. We also did not find a significant association between prostate cancer and testosterone. Androgen deprivation therapy (ADT) is the mainstay of prostate cancer treatment; however, recent research suggests that there are limits to androgen-related growth^69,70^. Future testosterone GWAS should continue to explore the potential link between testosterone and dementia and prostate cancer, which may involve changes in androgen receptor signaling rather than androgen levels.

A much higher proportion of men with hypogonadism were identified in the MVP (25%) compared to the UK Biobank (0.09%). As a result, we were well-powered to identify significant associations. The high incidence of hypogonadism in MVP may be partially related to the older age of the cohort (64 [18–110] years) compared to the UK Biobank (58 [37–73] years). We found that total testosterone levels decrease with age, with men older than 90 years of age having average total testosterone levels < 300 ng/dl (the threshold for hypogonadism diagnosis). Additionally, it may be related to the higher proportion of chronic disorders that can impact testosterone levels among Veterans, such as diabetes, lipid disorders, and liver disease. Selection bias could also contribute as all participants within the UK Biobank received testosterone testing upon enrollment, while MVP participants only received endocrine testing when ordered within a clinical setting.

The MVP provides a breadth of clinical and genomic data that has allowed us to conduct the most ancestrally diverse genetic analysis of testosterone, SHBG, and hypogonadism to date. However, our study has several limitations. First, as noted above, men participating in the MVP only had testosterone and/or SHBG levels measured when ordered by a physician, likely resulting in a selection bias for individuals already experiencing androgen-related symptoms. We also note limitations with exploring ancestry-specific mechanisms because the available tissue-specific gene expression data is predominantly derived from individuals with EUR ancestry. Thus, our colocalization analyses rely on EUR gene expression data even though gene expression patterns may be influenced by ancestry-specific effects. Furthermore, our GS analysis focuses on phecodes. These clinical outcomes are important for assessing disease risk; however, testosterone can also impact disease severity, treatment response, and disease progression.

Given that over 20% of men over 60 years of age have low testosterone levels^10^, this study may help improve clinical care by elucidating the genetic regulation of testosterone and its implication in disease. The Testosterone Trials previously examined the effects of testosterone treatment in older men^71^ but were not informed by androgen genetics. Genetic determinants of testosterone levels and cellular actions may provide important insight into testosterone replacement therapy (TRT) action and therapeutic benefit, as well as a greater understanding of androgen effects of testosterone on metabolic, cardiovascular, hepatic, and cognitive function. Moreover, this study deepens our understanding of testosterone genetic regulation and the role of genetically determined testosterone in hypogonadism, diabetes, liver disease, and dementia. Future directions should include the development of improved testosterone-relevant clinical phenotyping from electronic medical records, assessment of interactions between genomic determinants of testosterone and their impact on health and disease, and exploration of the links between testosterone genes and the functions of the liver, cardiovascular and metabolic systems, adrenal cortex, and brain.

## Methods

### Ethical approvals

All MVP participants provided informed consent upon enrollment. This study was approved by the VA Central Institutional Review Board and the Research & Development committees at the Salt Lake City VA and the San Diego VA.

### MVP Genotype quality

All study participants provided blood samples for DNA extraction and genotyping. Blood samples were collected by phlebotomists and banked at the VA Central Biorepository in Boston, MA, where DNA was extracted and shipped to 2 external centers for genotyping. DNA extracted from the buffy coat was genotyped using a custom Affymetrix Axiom Biobank array. The MVP 1.0 genotyping array contains a total of 723, 305 variants, enriched for low-frequency variants in African and Hispanic populations and variants associated with diseases common to the VA population^72,73^.

### Testosterone and hypogonadism phenotyping

We used the VA Corporate Data Warehouse (CDW) laboratory data to identify Veterans (*N* = 702,740) enrolled in the MVP from fiscal year 2011 to 2018. From the 702,740 Veterans, we selected Veterans who had documentation of at least one hormone laboratory test for testosterone (total testosterone, free testosterone, percent free testosterone, bioavailable testosterone, SHBG, and dihydrotestosterone) (*N* = 229,725). Among these 229,725, only 215,773 were male Veterans with documentation of at least one total testosterone laboratory value. From these 215,773, we excluded those with missing laboratory values (*N* = 886), incorrect units (e.g., mg/dL, ug/dL, %) (*N* = 7), inaccurate logical observation identifiers names and codes (LOINC) (*N* = 42), and a prescription for ADT before the total testosterone laboratory (*N* = 4769) or TRT (*N* = 6032), or had a diagnosis code for testicular cancer (*N* = 696) or orchiectomy (*N* = 168), resulting in a final analytical sample of 203,173 male Veterans (Fig. 1).

We excluded individuals on TRT and ADT to ensure we were assessing endogenous male sex hormone variation. Only morning levels from 7 AM to 12 PM were used. In individuals with multiple readings, the first measurement was used. Abnormally high levels (> 10,000 ng/dL) were removed. We only evaluated men, and patients with sex chromosome aneuploidy (XXY, XYY) were excluded from the analysis.

Individuals with either (1) a documented ICD hypogonadism code or (2) at least 2 low total testosterone values (< 300 ng/dL) were identified as hypogonadal. We selected individuals with no low testosterone readings, TRT, or ADT as matched controls. More detailed information on cohort selection is depicted in Supplementary Fig. S1.

### LDSC

Population-specific LD scores in EUR, AFR, AMR, and EAS ancestry groups were calculated for HapMap^74^ populations using intersecting variants from GWAS summary statistics and variant list (w_hm3.snplist, 1,217,312 variants). These scores were then used to calculate heritability estimates for total testosterone, free testosterone, SHBG, and hypogonadism for each ancestry group using LDSC v1.0.1 and default parameters.

### GWAS

Total testosterone, free testosterone, and SHBG levels (ng/dL) were inverse rank normalized. PLINK^75^ glm method was applied to conduct association analyses with testosterone levels by each ancestry group (EUR, AFR, AMR, EAS). Ancestry groups were determined by Wendt et al., which combined both a harmonized ancestry and race/ethnicity approach along with random-forest clustering using the 1000 Genomes Project and Human Genome Diversity Project^40^. Variants of minor allele frequency (MAF) less than 0.1% were excluded, and the first ten principal components of ancestry and age were used as covariates in the analysis. For hypogonadism analysis, the age of recorded hypogonadism diagnosis code or first age of low total testosterone levels (< 300 ng/dL) was used rather than the age of lab test^76^. In individuals with an ICD diagnosis code for hypogonadism and multiple low testosterone readings, the earliest age of hypogonadism identification was used. Significant and independent associations were identified with the linkage- and distance-based clumping method in PLINK using the GWAS significance threshold (*p* < 5 × 10^-8^). Variants within 500 kb and with *R*^2^ > 0.00001 were pruned away. A strict R^2^ threshold was used to prepare data for downstream Mendelian randomization results and to ensure independent loci. Alleles with significantly different (population-specific) effect sizes were identified via non-overlapping 90% confidence intervals between the sentinel ancestry and all other ancestries.

For EUR GWAS validation, we used UK Biobank summary statistics for European men (GCST90012103, GCST90012109, and GCST90012113) that were retrieved from Ruth et al.^26^ (https://www.ebi.ac.uk/gwas/publications/32042192#study_panel). For significant MVP variants not found in summary statistics, the closest variant (in base pairs) was used as a proxy. Proxy variants used for UK Biobank validation are provided in Supplementary Data 1 and 4. Novel variants were identified as those that met the GWAS significance threshold (*p* < 5 × 0−8) in MVP but not in the UK Biobank.

For AFR GWAS validation, total testosterone, free testosterone, and SHBG values were extracted and inverse-rank normalized for self-identifying African individuals from the Multi-Ethnic Study of Atherosclerosis (MESA). Individuals with KING relatedness > 0.177 were removed. Also, in cases of family genotypes, parents were excluded from analyses. The PLINK glm method was used to conduct validation analysis of testosterone loci in 1257 men. Age and the first ten principal components of ancestry were used as covariates. We excluded significant MVP total testosterone (1), free testosterone (1), SHBG (1), and hypogonadism (2) variants for the AFR ancestry group, which were not found in summary statistics were excluded from validation analysis.

### METAL

Meta-analysis of MVP EUR, AFR, AMR, and EAS GWAS with total testosterone levels, free testosterone levels, SHBG levels, and hypogonadism risk was conducted with METAL using standard error-based weights. Significant independent loci were identified through the linkage- and distance-based clumping method in PLINK and using GWAS significance threshold (*p* < 5 × 10^−8^). Variants within 500 kb and with *R*^2^ > 0.00001 were pruned away. Cochran’s heterogeneity analysis was conducted to identify variants with high variability between ancestry groups. Values are given in Supplementary Data 4 and plotted in Fig. 2.

### SuSiE Fine-mapping analysis

SuSiE requires a reference LD matrix and GWAS summary statistics. For each significant association, genotypes of all variants within 1 Mb were extracted from each MVP population, thinning individual count to 1000. Individual count had to be thinned to accommodate memory and computing parameters; moreover, LD may be accurately approximated using a subset of individuals from the GWAS cohort. Pairwise linkage disequilibrium was calculated for all variants within loci. For each phenotype (total testosterone, free testosterone, SHBG, hypogonadism), we calculated z-scores by dividing each effect size (beta) value by its standard error. Fine-mapping was run with SuSiE (susie.abf) using *L* = 10, which assumes a maximum of 10 causal variants. Matched reference populations were used for each analysis (e.g., EUR reference population was used for EUR ancestry associations). We focused on variants with a posterior inclusion probability of at least 10%.

### Gene mapping/colocalization analysis

#### To map variants to genes we used two approaches

1. Variant effect predictor (VEP) was run on all variants. Gene symbol output based on variant location is included in Supplementary Information. The majority of variants without annotation were intergenic or within regulatory regions.
2. Colocalization analysis using coloc R package^43^ (version v5.1.0) was used to determine the probability of shared causal variants between testosterone phenotypes and gene expression. We identified variants which were GTEx eQTLs in the European ancestry group (accession date: 10/02/2019) to assemble a gene set of interest. We then obtained full GTEx *cis-*eQTL association results for androgen-regulated tissues and/or sites (liver, testes, adrenal gland). The coloc.abf function was used to conduct analysis between GTEx *cis-*eQTL results and testosterone phenotype association analyses from the MVP EUR ancestry group. Genes with PP.H4 (posterior probability of one common causal variant between studies) of 0.8 between *cis-*eQTL and testosterone phenotype results were reported.

### Disease association analysis

We evaluated independent lead SNPs identified in the GWAS of free testosterone, hypogonadism, SHBG, and total testosterone for association to other diseases (i.e., PheWAS) in the un-analyzed portion of male MVP subjects (*n* = 545,764 for free testosterone, *n* = 448,328 for hypogonadism, *n* = 575,922 for SHBG, and *n* = 456,066 for total testosterone). PheWAS was conducted on 1875 phecode categories^77^ with the PheWAS R package^78^ (R version 4.0.3) by ancestry group using logistic regression. We restricted the analysis to the male sex and used age and the first ten genotyping principal components as covariates.

To confirm if variants from testosterone analyses shared causal variants with the disease, we extracted variants within a 100 Kb range and MAF of at least 0.1% for the variant of interest. We ran coloc^43^ R package (version v3.2.1) on variant *p*-values from total testosterone levels, free testosterone levels, SHBG levels, or hypogonadism GWAS analyses and disease PheWAS. Variant MAF from PheWAS analysis was used in coloc analysis.

### Mendelian randomization

TwoSampleMR (version 0.6.6)^45,46^ was run on significant associations for total testosterone, SHBG, and hypogonadism variants from testosterone meta-analysis and published GWAS studies for obesity, hyperlipidemia, gout, type 2 diabetes, and MASLD. Published GWAS details are given in Supplementary Data 7. Variants from MVP meta-analyses were harmonized with published GWAS information. No further clumping was performed as we used strict linkage disequilibrium thresholds to identify significant GWAS variants.

### GS and GRS Construction and disease association

We constructed GS/GRSs using only genome-wide significant meta-analysis variants (*p* < 5 × 10^−8^) for total testosterone, SHBG, and hypogonadism. Variants were extracted from imputed genotype BGEN files. Alleles were oriented to increasing testosterone or SHBG levels or increasing the risk of hypogonadism. Genotype dosages were weighted by effect size estimates for each respective ancestry group and summed. Validation of GS/GRS was conducted within discovery groups for each analysis.

Cox proportional hazards analyses were conducted using R survival package (***) in EUR, AFR, AMR, and EAS MVP groups with phecodes used in PheWAS analyses (see Disease Association Analysis) for event and age of diagnosis for time to event. Individuals included in discovery analyses, those with chromosomal abnormalities (XXY, XXX, XYY), and those on ADT or TRT were removed from analyses. Analyses were controlled for age of enrollment and the top 3 genotyping principal components as covariates.

### Reporting summary

Further information on research design is available in the Nature Portfolio Reporting Summary linked to this article.

## Supplementary information

Supplemental Information

Description of Additional Supplementary Files

Supplemental Dataset 1

Supplemental Dataset 2

Supplemental Dataset 3

Supplemental Dataset 4

Supplemental Dataset 5

Supplemental Dataset 6

Supplemental Dataset 7

Supplemental Dataset 8

Reporting Summary

Transparent Peer Review file

## Data Availability

The full summary-level association data generated in this study are deposited in the GWAS Catalog database https://www.ebi.ac.uk/gwas/ under accession numbers GCST90503313, GCST90503314, GCST90503315, GCST90503316, GCST90503317, GCST90503318, GCST90503319, GCST90503320, GCST90503321, GCST90503322, GCST90503323, GCST90503324, GCST90503325, GCST90503326, GCST90503327, GCST90503328. The source data generated in this study are provided in the Supplementary Datasets provided in this paper.

## Code availability

Code to reproduce manuscript figures is available at: https://github.com/meghatron21/mvp-testosterone-gwas.

## References

[1] Bhasin, S. et al. Testosterone therapy in men with hypogonadism: An endocrine society clinical practice guideline. *J. Clin. Endocrinol. Metab.* **103**, 1715–1744 (2018). 29562364 10.1210/jc.2018-00229

[2] Matsumoto, A. M. & Bremner, W. J. in *Williams Textbook of Endocrinology* 688–777 (Elsevier, 2011).

[3] Wilson, D. J., Griffin, E., WGeorge, J. F. & Leshin, M. The endocrine control of male phenotypic development. *Aust. J. Biol. Sci.* **36**, 101 (1983). 6354161 10.1071/bi9830101

[4] Shahani, S., Braga-Basaria, M., Maggio, M. & Basaria, S. Androgens and erythropoiesis: past and present. *J. Endocrinol. Invest.* **32**, 704–716 (2009). 19494706 10.1007/BF03345745

[5] Herbst, K. L. & Bhasin, S. Testosterone action on skeletal muscle. *Curr. Opin. Clin. Nutr. Metab. Care* **7**, 271–277 (2004). 15075918 10.1097/00075197-200405000-00006

[6] Gubbels Bupp, M. R. & Jorgensen, T. N. Androgen-induced immunosuppression. *Front. Immunol.* **9**, 794 (2018). 29755457 10.3389/fimmu.2018.00794 PMC5932344

[7] Kelly, D. M. & Jones, T. H. Testosterone: a vascular hormone in health and disease. *J. Endocrinol.* **217**, R47–R71 (2013). 23549841 10.1530/JOE-12-0582

[8] O’Reilly, M. W., House, P. J. & Tomlinson, J. W. Understanding androgen action in adipose tissue. *J. Steroid Biochem. Mol. Biol.* **143**, 277–284 (2014). 24787657 10.1016/j.jsbmb.2014.04.008

[9] Davey, R. A. & Grossmann, M. Androgen receptor structure, function and biology: From bench to bedside. *Clin. Biochem. Rev.* **37**, 3–15 (2016). 27057074 PMC4810760

[10] Kaufman, J. M. & Vermeulen, A. The decline of androgen levels in elderly men and its clinical and therapeutic implications. *Endocr. Rev.* **26**, 833–876 (2005). 15901667 10.1210/er.2004-0013

[11] Kaufman, J.-M., Lapauw, B., Mahmoud, A., T’Sjoen, G. & Huhtaniemi, I. T. Aging and the male reproductive system. *Endocr. Rev.* **40**, 906–972 (2019). 30888401 10.1210/er.2018-00178

[12] Huhtaniemi, I. & Forti, G. Male late-onset hypogonadism: pathogenesis, diagnosis and treatment. *Nat. Rev. Urol.* **8**, 335–344 (2011). 21502974 10.1038/nrurol.2011.47

[13] Zhao, J. V. & Schooling, C. M. The role of testosterone in chronic kidney disease and kidney function in men and women: a bi-directional Mendelian randomization study in the UK Biobank. *BMC Med.* **18**, 122 (2020). 32493397 10.1186/s12916-020-01594-x PMC7271464

[14] Yeap, B. B. et al. Lower testosterone levels predict incident stroke and transient ischemic attack in older men. *J. Clin. Endocrinol. Metab.* **94**, 2353–2359 (2009). 19351733 10.1210/jc.2008-2416

[15] Ohlsson, C. et al. High serum testosterone is associated with reduced risk of cardiovascular events in elderly men. The MrOS (Osteoporotic Fractures in Men) study in Sweden. *J. Am. Coll. Cardiol.* **58**, 1674–1681 (2011). 21982312 10.1016/j.jacc.2011.07.019

[16] Yeap, B. B. et al. In older men an optimal plasma testosterone is associated with reduced all-cause mortality and higher dihydrotestosterone with reduced ischemic heart disease mortality, while estradiol levels do not predict mortality. *J. Clin. Endocrinol. Metab.* **99**, E9–E18 (2014). 24257908 10.1210/jc.2013-3272

[17] Laughlin, G. A., Barrett-Connor, E. & Bergstrom, J. Low serum testosterone and mortality in older men. *J. Clin. Endocrinol. Metab.* **93**, 68–75 (2008). 17911176 10.1210/jc.2007-1792 PMC2190742

[18] Haring, R. et al. Low serum testosterone levels are associated with increased risk of mortality in a population-based cohort of men aged 20-79. *Eur. Heart J.* **31**, 1494–1501 (2010). 20164245 10.1093/eurheartj/ehq009

[19] Wittert, G. et al. Testosterone treatment to prevent or revert type 2 diabetes in men enrolled in a lifestyle programme (T4DM): a randomised, double-blind, placebo-controlled, 2-year, phase 3b trial. *Lancet Diab. Endocrinol.* **9**, 32–45 (2021). 10.1016/S2213-8587(20)30367-3 33338415

[20] Nead, K. T. Androgens and depression: a review and update. *Curr. Opin. Endocrinol. Diab. Obes.* **26**, 175–179 (2019). 10.1097/MED.0000000000000477 30958398

[21] Panizzon, M. S. et al. Interaction of APOE genotype and testosterone on episodic memory in middle-aged men. *Neurobiol. Aging* **35**, 1778.e1–8 (2014). 24444806 10.1016/j.neurobiolaging.2013.12.025 PMC3980008

[22] Vegeto, E. et al. The role of sex and sex hormones in neurodegenerative diseases. *Endocr. Rev.* **41**, 273–319 (2020). 31544208 10.1210/endrev/bnz005 PMC7156855

[23] Vest, R. S. & Pike, C. J. Gender, sex steroid hormones, and Alzheimer’s disease. *Horm. Behav.* **63**, 301–307 (2013). 22554955 10.1016/j.yhbeh.2012.04.006 PMC3413783

[24] Yeap, B. B. Hormonal changes and their impact on cognition and mental health of ageing men. *Maturitas* **79**, 227–235 (2014). 24953176 10.1016/j.maturitas.2014.05.015

[25] Hauger, R. L., Saelzler, U. G., Pagadala, MeghanaS. & Panizzon, M. S. The role of testosterone, the androgen receptor, and hypothalamic-pituitary-gonadal axis in depression in ageing Men. *Rev. Endocr. Metab. Disord.* **23**, 1259–1273 (2022). 36418656 10.1007/s11154-022-09767-0 PMC9789012

[26] Ruth, K. S. et al. Using human genetics to understand the disease impacts of testosterone in men and women. *Nat. Med.* **26**, 252–258 (2020). 32042192 10.1038/s41591-020-0751-5 PMC7025895

[27] Sinnott-Armstrong, N., Naqvi, S., Rivas, M. & Pritchard, J. K. GWAS of three molecular traits highlights core genes and pathways alongside a highly polygenic background. *Elife* **10**, 10.7554/elife.58615 (2021). 10.7554/eLife.58615 PMC7884075 33587031

[28] Prins, B. P. et al. Genome-wide analysis of health-related biomarkers in the UK Household Longitudinal Study reveals novel associations. *Sci. Rep.* **7**, 11008 (2017). 28887542 10.1038/s41598-017-10812-1 PMC5591265

[29] Flynn, E. et al. Sex-specific genetic effects across biomarkers. *Eur. J. Hum. Genet.* **29**, 154–163 (2021). 32873964 10.1038/s41431-020-00712-w PMC7794464

[30] Jin, G. et al. Genome-wide association study identifies a new locus JMJD1C at 10q21 that may influence serum androgen levels in men. *Hum. Mol. Genet.* **21**, 5222–5228 (2012). 22936694 10.1093/hmg/dds361 PMC3607470

[31] Ohlsson, C. et al. Genetic determinants of serum testosterone concentrations in men. *PLoS Genet.* **7**, e1002313 (2011). 21998597 10.1371/journal.pgen.1002313 PMC3188559

[32] Chen, Z. et al. Genome-wide association study of sex hormones, gonadotropins and sex hormone-binding protein in Chinese men. *J. Med. Genet.* **50**, 794–801 (2013). 24049095 10.1136/jmedgenet-2013-101705

[33] Leinonen, J. T. et al. Genetic analyses implicate complex links between adult testosterone levels and health and disease. *Commun. Med.* **3**, 4 (2023). 36653534 10.1038/s43856-022-00226-0 PMC9849476

[34] Fantus, R. J. et al. Genetic susceptibility for low testosterone in men and its implications in biology and screening: Data from the UK Biobank. *Eur. Urol. Open Sci.* **29**, 36–46 (2021). 34337532 10.1016/j.euros.2021.04.010 PMC8317803

[35] Ellis, L. & Nyborg, H. Racial/ethnic variations in male testosterone levels: a probable contributor to group differences in health. *Steroids* **57**, 72–75 (1992). 1621259 10.1016/0039-128x(92)90032-5

[36] Lopez, D. S. et al. Racial/ethnic differences in serum sex steroid hormone concentrations in US adolescent males. *Cancer Causes Control* **24**, 817–826 (2013). 23354421 10.1007/s10552-013-0154-8 PMC3850289

[37] Nyante, S. J. et al. Trends in sex hormone concentrations in US males: 1988-1991 to 1999-2004. *Int. J. Androl.* **35**, 456–466 (2012). 22150314 10.1111/j.1365-2605.2011.01230.x PMC4137971

[38] Feldman, H. A. et al. Age trends in the level of serum testosterone and other hormones in middle-aged men: longitudinal results from the Massachusetts male aging study. *J. Clin. Endocrinol. Metab.* **87**, 589–598 (2002). 11836290 10.1210/jcem.87.2.8201

[39] Aribas, E., Kavousi, M., Laven, J. S. E., Ikram, M. A. & van Lennep, J. E. Aging, cardiovascular risk, and SHBG levels in men and women from the general population. *J. Clin. Endocrinol. Metab.* **106**, 2890–2900 (2021). 34197576 10.1210/clinem/dgab470 PMC8475196

[40] Wendt, F. R. et al. Modeling the longitudinal changes of ancestry diversity in the Million Veteran Program. *Hum. Genom.* **17**, 46 (2023). 10.1186/s40246-023-00487-3 PMC10239111 37268996

[41] Finucane, H. K. et al. Partitioning heritability by functional annotation using genome-wide association summary statistics. *Nat. Genet.* **47**, 1228–1235 (2015). 26414678 10.1038/ng.3404 PMC4626285

[42] Benner, C. et al. FINEMAP: efficient variable selection using summary data from genome-wide association studies. *Bioinformatics* **32**, 1493–1501 (2016). 26773131 10.1093/bioinformatics/btw018 PMC4866522

[43] Giambartolomei, C. et al. Bayesian test for colocalisation between pairs of genetic association studies using summary statistics. *PLoS Genet.* **10**, e1004383 (2014). 24830394 10.1371/journal.pgen.1004383 PMC4022491

[44] Bycroft, C. et al. The UK Biobank resource with deep phenotyping and genomic data. *Nature* **562**, 203–209 (2018). 30305743 10.1038/s41586-018-0579-z PMC6786975

[45] Hemani, G., Tilling, K. & Davey Smith, G. Orienting the causal relationship between imprecisely measured traits using GWAS summary data. *PLoS Genet.* **13**, e1007081 (2017). 29149188 10.1371/journal.pgen.1007081 PMC5711033

[46] Hemani, G. et al. The MR-Base platform supports systematic causal inference across the human phenome. *Elife* **7**, 10.7554/elife.34408 (2018). 10.7554/eLife.34408 PMC5976434 29846171

[47] Karthikeyan, G. et al. Lipid profile, plasma apolipoproteins, and risk of a first myocardial infarction among Asians: an analysis from the INTERHEART Study. *J. Am. Coll. Cardiol.* **53**, 244–253 (2009). 19147041 10.1016/j.jacc.2008.09.041

[48] Frank, A. T. H. et al. Racial/ethnic differences in dyslipidemia patterns. *Circulation* **129**, 570–579 (2014). 24192801 10.1161/CIRCULATIONAHA.113.005757 PMC4212818

[49] Truesdale, K. P., Stevens, J. & Cai, J. Impact of body mass index levels on lipid abnormalities in Chinese Asians, American Blacks and American Whites: the People’s Republic of China (PRC) and Atherosclerosis Risk in Communities (ARIC) Studies. *Atherosclerosis* **218**, 517–523 (2011). 21802083 10.1016/j.atherosclerosis.2011.06.052 PMC3234685

[50] Goff, D. C. Jr et al. Dyslipidemia prevalence, treatment, and control in the Multi-Ethnic Study of Atherosclerosis (MESA): gender, ethnicity, and coronary artery calcium. *Circulation* **113**, 647–656 (2006). 16461837 10.1161/CIRCULATIONAHA.105.552737

[51] Vujkovic, M. et al. A multiancestry genome-wide association study of unexplained chronic ALT elevation as a proxy for nonalcoholic fatty liver disease with histological and radiological validation. *Nat. Genet.* **54**, 761–771 (2022). 35654975 10.1038/s41588-022-01078-z PMC10024253

[52] Fawcett, K. A. et al. Pleiotropic associations of heterozygosity for the SERPINA1 Z allele in the UK Biobank. *ERJ Open Res.* **7**, 49–2021 (2021). 10.1183/23120541.00049-2021 PMC8107350 33981765

[53] Hamesch, K. et al. Liver fibrosis and metabolic alterations in adults with alpha-1-antitrypsin deficiency caused by the Pi*ZZ mutation. *Gastroenterology* **157**, 705–719 (2019). 31121167 10.1053/j.gastro.2019.05.013

[54] Strnad, P. et al. Heterozygous carriage of the alpha1-antitrypsin Pi*Z variant increases the risk to develop liver cirrhosis. *Gut* **68**, 1099–1107 (2019). 30068662 10.1136/gutjnl-2018-316228

[55] Zhou, B. et al. The nuclear orphan receptor NR2F6 promotes hepatic steatosis through upregulation of fatty acid transporter CD36. *Adv. Sci.* **7**, 2002273 (2020). 10.1002/advs.202002273 PMC7610302 33173745

[56] Fernandes Silva, L., Vangipurapu, J., Kuulasmaa, T. & Laakso, M. An intronic variant in the GCKR gene is associated with multiple lipids. *Sci. Rep.* **9**, 10240 (2019). 31308433 10.1038/s41598-019-46750-3 PMC6629684

[57] Sargsyan, A. et al. HGFAC is a ChREBP-regulated hepatokine that enhances glucose and lipid homeostasis. *JCI Insight* **8**, 10.1172/jci.insight.153740 (2023). 10.1172/jci.insight.153740 PMC9870088 36413406

[58] Chen, V. L., Chen, Y., Du, X., Handelman, S. K. & Speliotes E. K. Genetic variants that associate with cirrhosis have pleiotropic effects on human traits. *Liver Int.* **40**, 405–415 (2020). 31815349 10.1111/liv.14321 PMC7395656

[59] Pazoki, R. et al. Genetic analysis in European ancestry individuals identifies 517 loci associated with liver enzymes. *Nat. Commun.* **12**, 2579 (2021). 33972514 10.1038/s41467-021-22338-2 PMC8110798

[60] Schonfeld, M., Villar, M. T., Artigues, A., Weinman, S. A. & Tikhanovich, I. Arginine methylation of integrin alpha-4 prevents fibrosis development in alcohol-associated liver disease. *Cell. Mol. Gastroenterol. Hepatol.* **15**, 39–59 (2023). 36191854 10.1016/j.jcmgh.2022.09.013 PMC9672451

[61] Scaramuzzino, C. et al. Protein arginine methyltransferase 6 enhances polyglutamine-expanded androgen receptor function and toxicity in spinal and bulbar muscular atrophy. *Neuron* **85**, 88–100 (2015). 25569348 10.1016/j.neuron.2014.12.031 PMC4305189

[62] Grossmann, M. et al. Low testosterone levels are common and associated with insulin resistance in men with diabetes. *J. Clin. Endocrinol. Metab.* **93**, 1834–1840 (2008). 18319314 10.1210/jc.2007-2177

[63] Panizzon, M. S. et al. Testosterone modifies the effect of APOE genotype on hippocampal volume in middle-aged men. *Neurology* **75**, 874–880 (2010). 20819998 10.1212/WNL.0b013e3181f11deb PMC2938973

[64] Moffat, S. D. et al. Longitudinal assessment of serum free testosterone concentration predicts memory performance and cognitive status in elderly men. *J. Clin. Endocrinol. Metab.* **87**, 5001–5007 (2002). 12414864 10.1210/jc.2002-020419

[65] Asih, P. R. et al. Testosterone replacement therapy in older male subjective memory complainers: double-blind randomized crossover placebo-controlled clinical trial of physiological assessment and safety. *CNS Neurol. Disord. Drug Targets* **14**, 576–586 (2015). 25921747 10.2174/1871527314666150429112112

[66] Lv, W. et al. Low testosterone level and risk of Alzheimer’s disease in the elderly men: A systematic review and meta-analysis. *Mol. Neurobiol.* **53**, 2679–2684 (2016). 26154489 10.1007/s12035-015-9315-y

[67] Rosario, E. R. & Pike, C. J. Androgen regulation of beta-amyloid protein and the risk of Alzheimer’s disease. *Brain Res. Rev.* **57**, 444–453 (2008). 17658612 10.1016/j.brainresrev.2007.04.012 PMC2390933

[68] Lu, Y. et al. Low-level laser therapy for beta amyloid toxicity in rat hippocampus. *Neurobiol. Aging* **49**, 165–182 (2017). 27815990 10.1016/j.neurobiolaging.2016.10.003 PMC5458630

[69] Morgentaler, A. & Traish, A. M. Shifting the paradigm of testosterone and prostate cancer: the saturation model and the limits of androgen-dependent growth. *Eur. Urol.* **55**, 310–320 (2009). 18838208 10.1016/j.eururo.2008.09.024

[70] Prostate Cancer Collaborative Endogenous Hormones Group, Roddam, A. W., Allen, N. E., Appleby, P. & Key, T. J. Endogenous sex hormones and prostate cancer: a collaborative analysis of 18 prospective studies. *J. Natl. Cancer Inst.* **100**, 170–183 (2008). 18230794 10.1093/jnci/djm323 PMC6126902

[71] Snyder, P. J. et al. Lessons from the testosterone trials. *Endocr. Rev.* **39**, 369–386 (2018). 29522088 10.1210/er.2017-00234 PMC6287281

[72] Gaziano, J. M. et al. Million Veteran Program: A mega-biobank to study genetic influences on health and disease. *J. Clin. Epidemiol.* **70**, 214–223 (2016). 26441289 10.1016/j.jclinepi.2015.09.016

[73] Hunter-Zinck, H. et al. Genotyping array design and data quality control in the Million Veteran Program. *Am. J. Hum. Genet.* **106**, 535–548 (2020). 32243820 10.1016/j.ajhg.2020.03.004 PMC7118558

[74] International HapMap 3 Consortium et al. Integrating common and rare genetic variation in diverse human populations. *Nature* **467**, 52–58 (2010). 10.1038/nature09298 PMC3173859 20811451

[75] Purcell, S. et al. PLINK: a tool set for whole-genome association and population-based linkage analyses. *Am. J. Hum. Genet.* **81**, 559–575 (2007). 17701901 10.1086/519795 PMC1950838

[76] Mulhall, J. P. et al. Evaluation and management of testosterone deficiency: AUA guideline. *J. Urol.* **200**, 423–432 (2018). 29601923 10.1016/j.juro.2018.03.115

[77] Wu, P. et al. Mapping ICD-10 and ICD-10-CM codes to phecodes: Workflow development and initial evaluation. *JMIR Med. Inform.* **7**, e14325 (2019). 31553307 10.2196/14325 PMC6911227

[78] Carroll, R. J., Bastarache, L. & Denny, J. C. R. PheWAS: data analysis and plotting tools for phenome-wide association studies in the R environment. *Bioinformatics* **30**, 2375–2376 (2014). 24733291 10.1093/bioinformatics/btu197 PMC4133579

