# Supplementary material for: Discovery of novel ancestry specific genes for androgens and hypogonadism in Million Veteran Program Men
Source: Nat Commun. 2025 May 2;16:4104. doi: 10.1038/s41467-025-57372-x (PMC12048691; doi:10.1038/s41467-025-57372-x)

**Table S1: LDSC Heritability Estimates for Total Testosterone, Free Testosterone, SHBG levels and Hypogonadism**

|  | Total | Free | SHBG | Hypogonad |
| --- | --- | --- | --- | --- |
| EUR | .10 (.02) | .02 (.01) | .20 (.05) | .08 (.01) |
| AFR | .12 (.03) | -0.04 (0.07) | .5 (.29) | .05 (.03) |
| AMR | .09 (.04) | -0.02 (.08) | .43 (.24) | .09 (.04) |
| EAS | NA | NA | NA | NA |

**Table S2: LDSC Genetic Correlation Estimates for Total Testosterone, Free Testosterone, SHBG levels and Hypogonadism**

| **EUR** | Total | Free | SHBG | Hypogonad |
| --- | --- | --- | --- | --- |
| Total | 1 | 0.61 (0.21) | 0.71 (0.09) | -0.97 (0.03) |
| Free |  | 1 | 0.13 (0.23) | -0.63 (0.23) |
| SHBG |  |  | 1 | -0.73 (0.10) |
| Hypogonad |  |  |  | 1 |
|  |  |  |  |  |
| **AFR** | Total | Free | SHBG | Hypogonad |
| Total | 1 | NA | 0.45 (0.24) | -0.90 (0.21) |
| Free |  | 1 | NA | NA |
| SHBG |  |  | 1 | -0.51 (0.37) |
| Hypogonad |  |  |  | 1 |
|  |  |  |  |  |
| **AMR** | Total | Free | SHBG | Hypogonad |
| Total | 1 | NA | 0.97 (0.41) | -0.88 (0.21) |
| Free |  | 1 | NA | NA |
| SHBG |  |  | 1 | 0.19 (0.37) |
| Hypogonad |  |  |  | 1 |
|  |  |  |  |  |
| **EAS** | Total | Free | SHBG | Hypogonad |
| Total | 1 | NA | NA | NA |
| Free |  | 1 | NA | NA |
| SHBG |  |  | 1 | NA |
| Hypogonad |  |  |  | 1 |

**Figure S1: Workflow of Testosterone and Hypogonadism Phenotype Processing in MVP.** Steps to process testosterone information from MVP. Individuals on ADT and TRT. Analysis was focused on morning (7 AM – 12 PM) testosterone levels. Related individuals and individuals with sex aneuploidies were excluded. Hypogonadism cases were based on 1) hypogonadism code and 2) at least 2 low testosterone readings (< 300 ng/dl). Hypogonadism controls for analysis included individuals not on ADT nor TRT without a low testosterone reading.

**
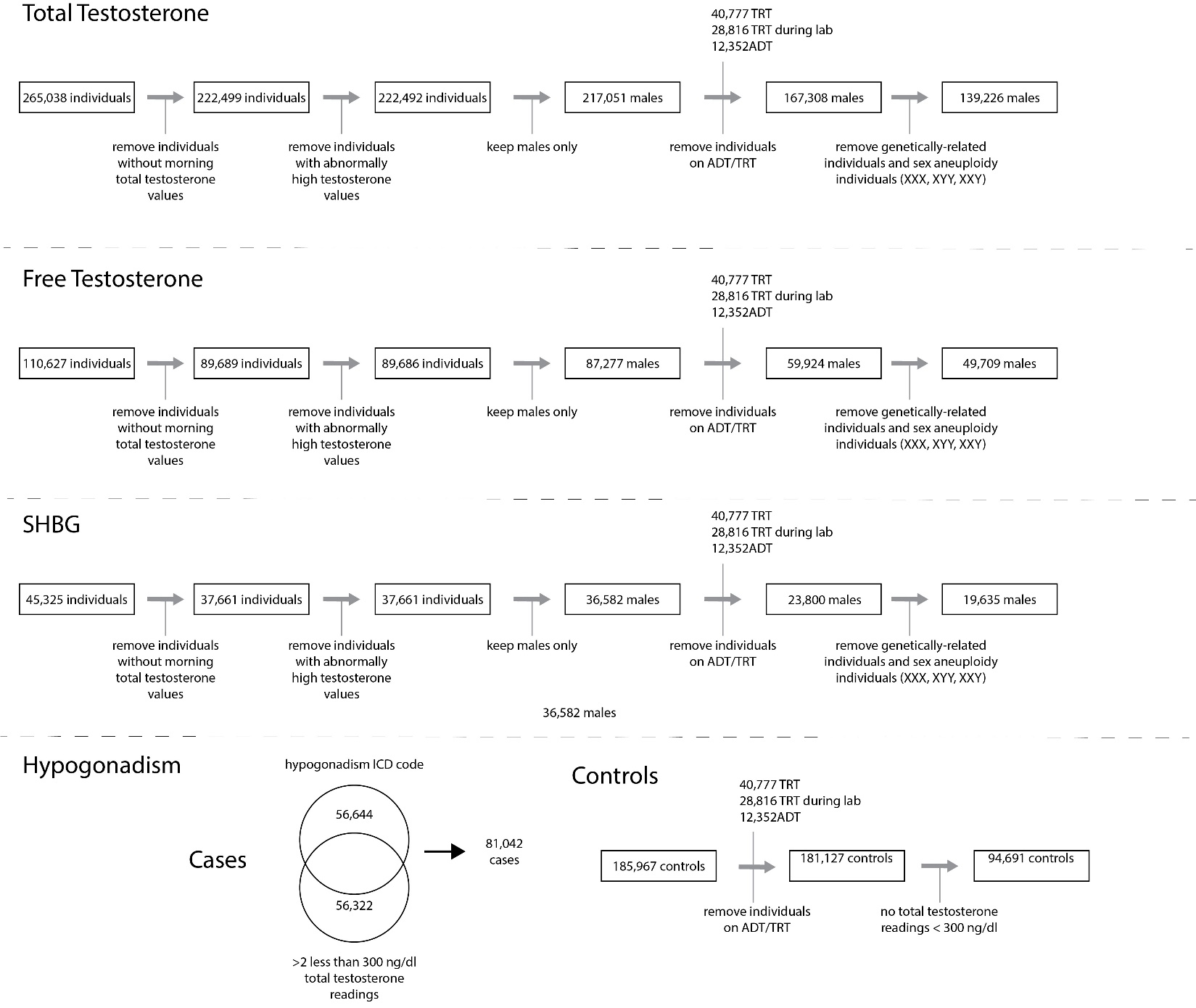
**

**Figure S2: Testosterone and Hypogonadism Phenotyping in MVP. (A)** Histogram of total testosterone **(A)**, free testosterone **(B)** and SHBG levels **(C)** across all ancestry groups**.** Decile plot of total testosterone **(D),** free testosterone **(E),** and SHBG **(F)** levels by age of lab test across all ancestry groups. Boxplot of total testosterone (**G),** free testosterone **(H),** and SHBG levels **(I)** by EUR, AFR, AMR, EAS ancestry group. **(J)** Incidence of hypogonadism by EUR, AFR, AMR, EAS ancestry group. Individuals were identified with hypogonadism if reported hypogonadism diagnosis or at least 2 low total testosterone readings (<300 ng/dL). **(K)** Density plot of total testosterone levels in individuals with documented hypogonadism diagnosis, at least 2 low total testosterone levels (<300 ng/dL) and matched controls.


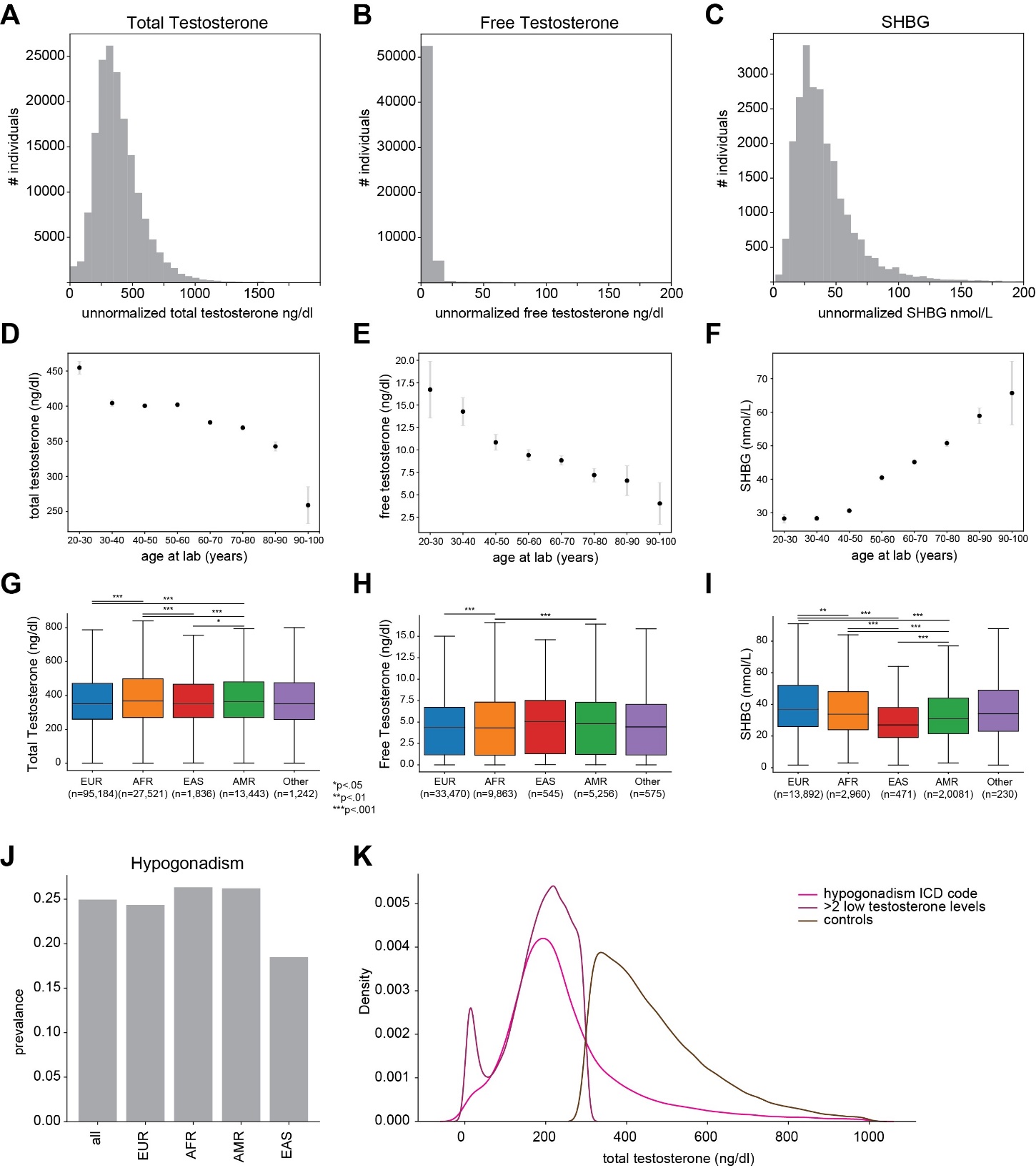


**Figure S3: Ancestry Group GWAS with Total Testosterone, Free Testosterone, SHBG and Hypogonadism.** QQ and Manhattan plots of total testosterone **(A-D),** free testosterone (**E-H),** SHBG (**I-L),** and hypogonadism (**M-P)** GWAS for EUR, AFR, AMR and EAS genetic ancestry groups.


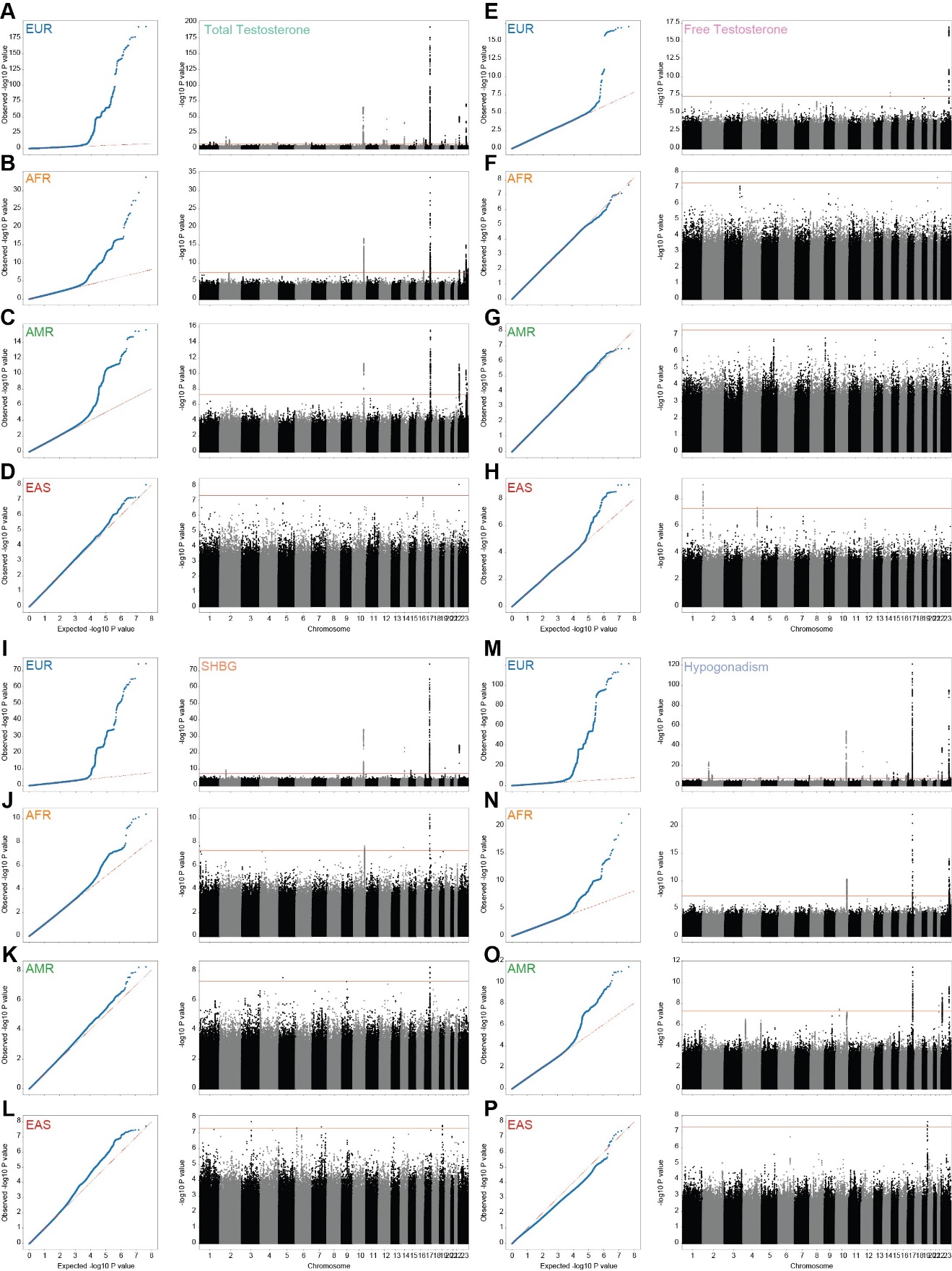


**Figure S4: Variant Effect Sizes from MVP Ancestry Group GWAS.** Plots of ancestry GWAS with significant effect size differences (no overlap of 90% confidence interval of beta) **(A).** METAL trans-ancestry SNPs with significant heterogeneity scores **(B)** and non-significant heterogeneity scores (**C).**

**
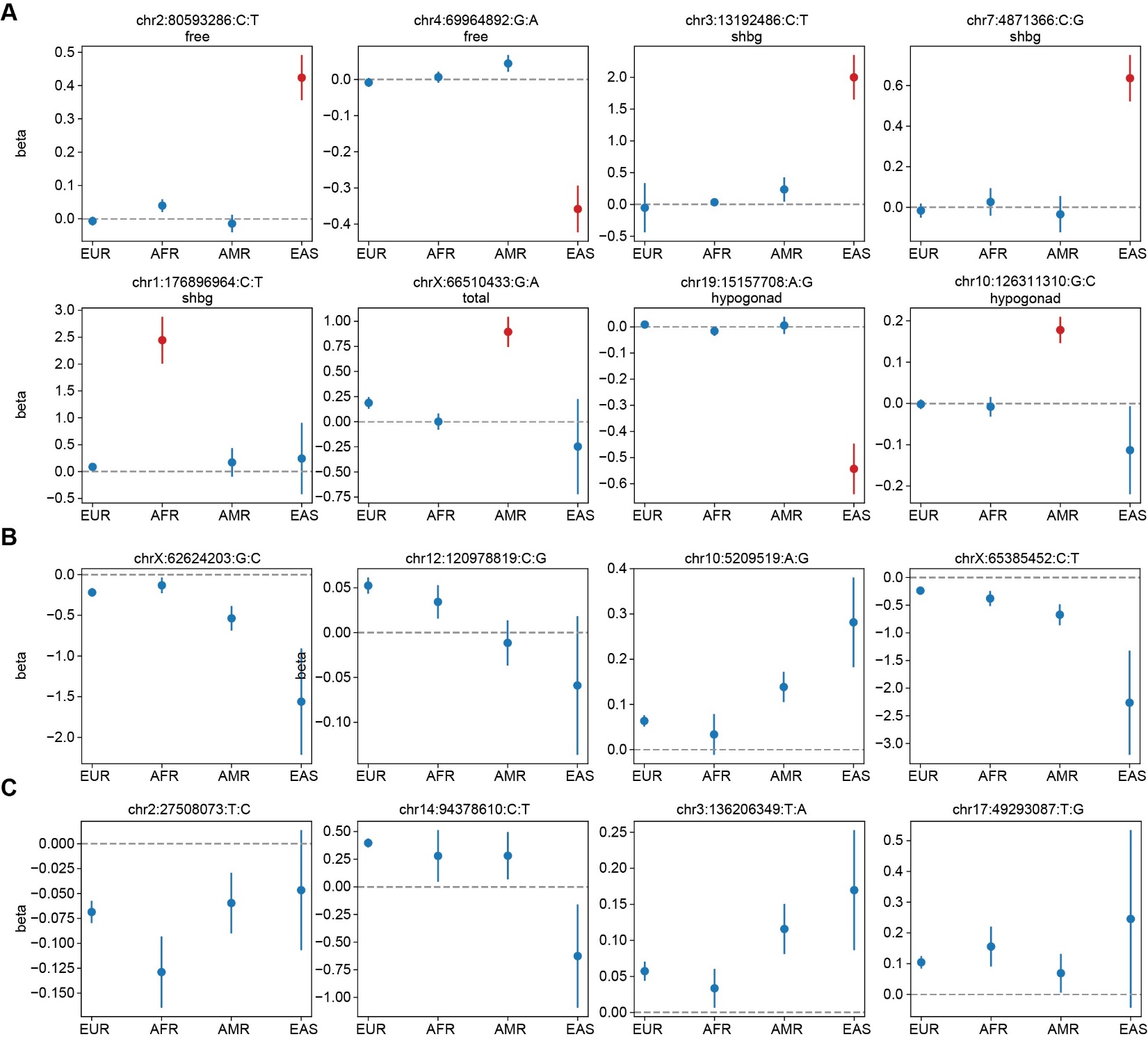
**

**Figure S5: Analysis of Minor Allele Frequencies (MAFs) and Effect Sizes of MVP Variants Not Validated in UK Biobank. (A)** Scatterplot of MVP and UK Biobank effect sizes (beta) for total and free testosterone associations significant in MVP but not UK Biobank. **(B)** Barplot of UK Biobank and MVP MAF for six variants significant in MVP but not UK Biobank.


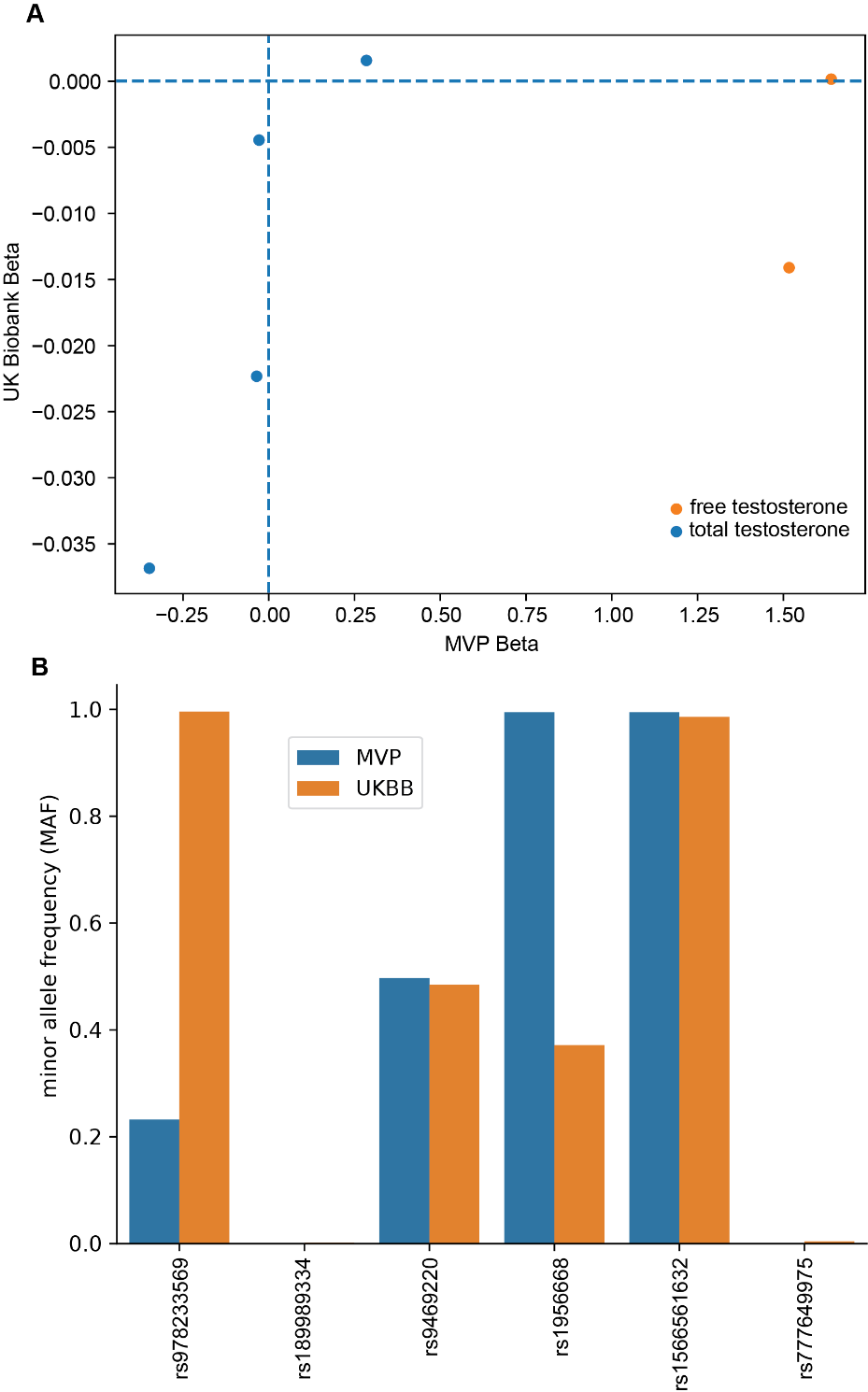


**Figure S6: Validation of MVP AFR GWAS variants in MESA cohort.** Scatterplot of MVP and MESA Testosterone GWAS beta and standard error values in only AFR men. No points pass significance threshold p<.001.


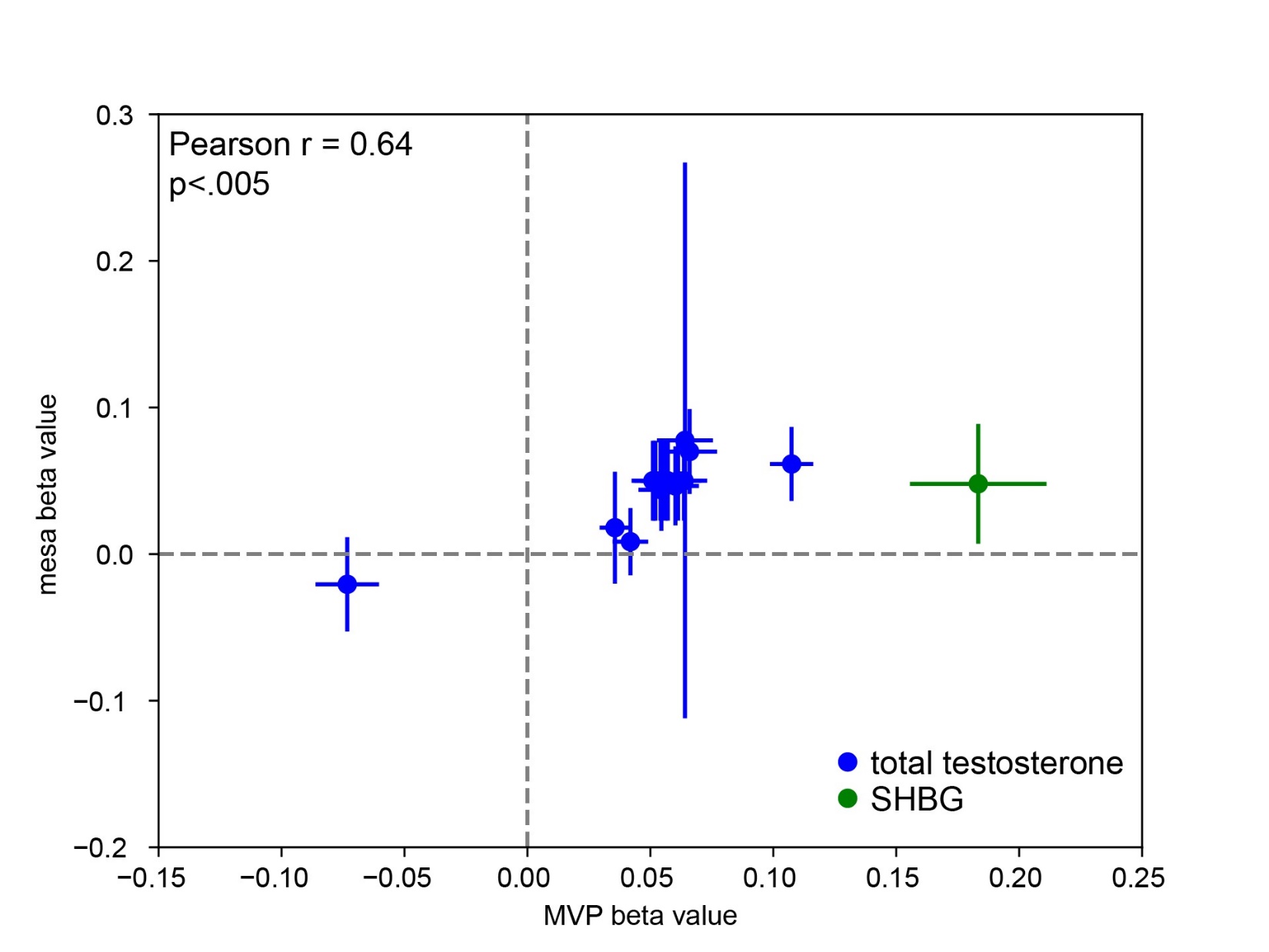


**Figure S7: Effects of Total Testosterone, SHBG, and Hypogonadism Risk Variants Identified through METAL Analysis Across MVP Ancestry Groups.** Clustermap of total testosterone **(A),** free testosterone **(B),** SHBG **(C)** and hypogonadism **(D)** variant effect sizes (beta) for EUR, AFR, AMR, and EAS ancestry groups.


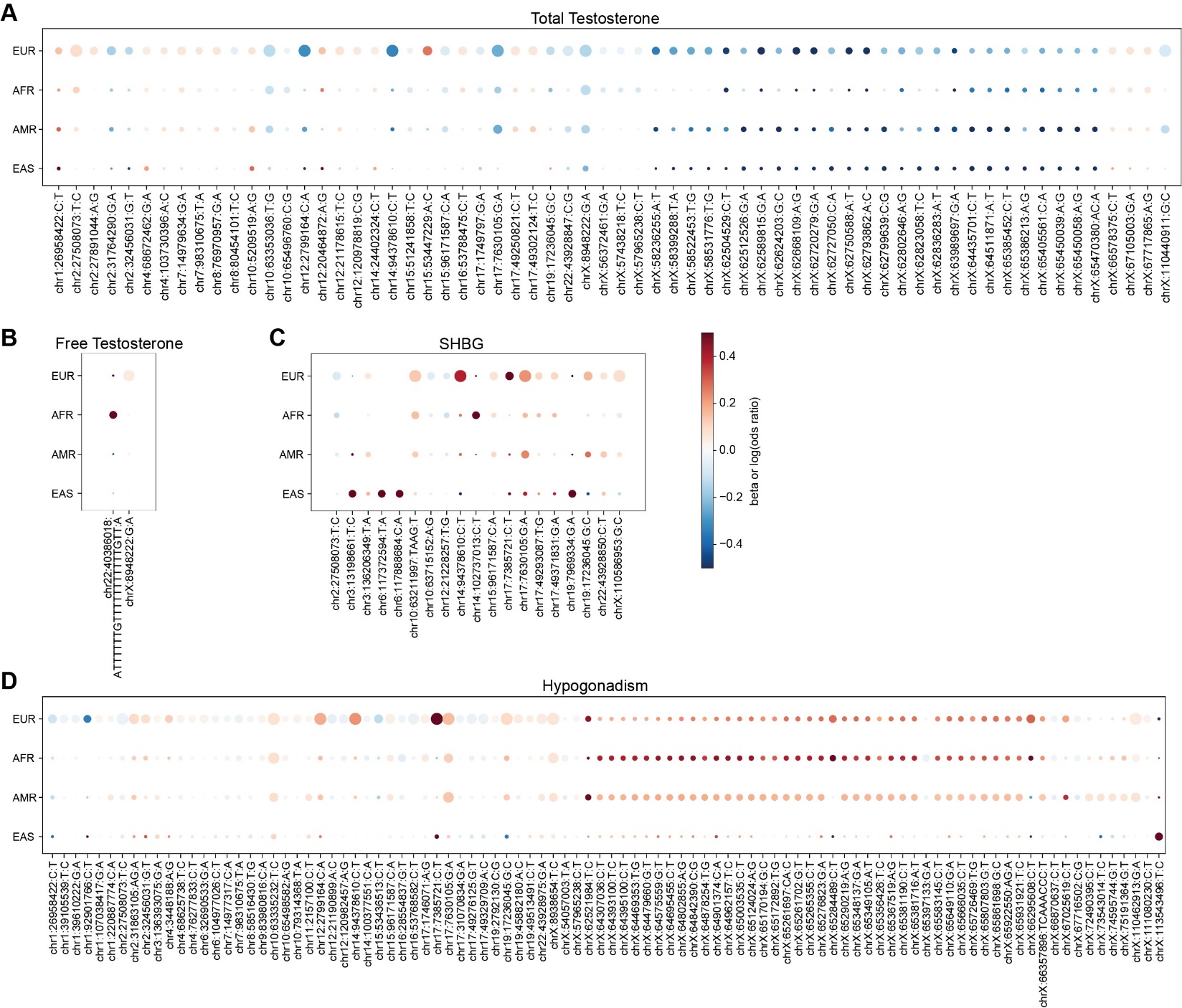


**Figure S8: Validation of Total Testosterone, SHBG GS and Hypogonadism GRS in UK Biobank.** Decile plots of total testosterone **(A),** SHBG **(B)** GS and hypogonadism **(C)** GRS and corresponding levels of total testosterone (ng/dL), SHBG levels (nmol/dl) and odds of hypogonadism, respectively.


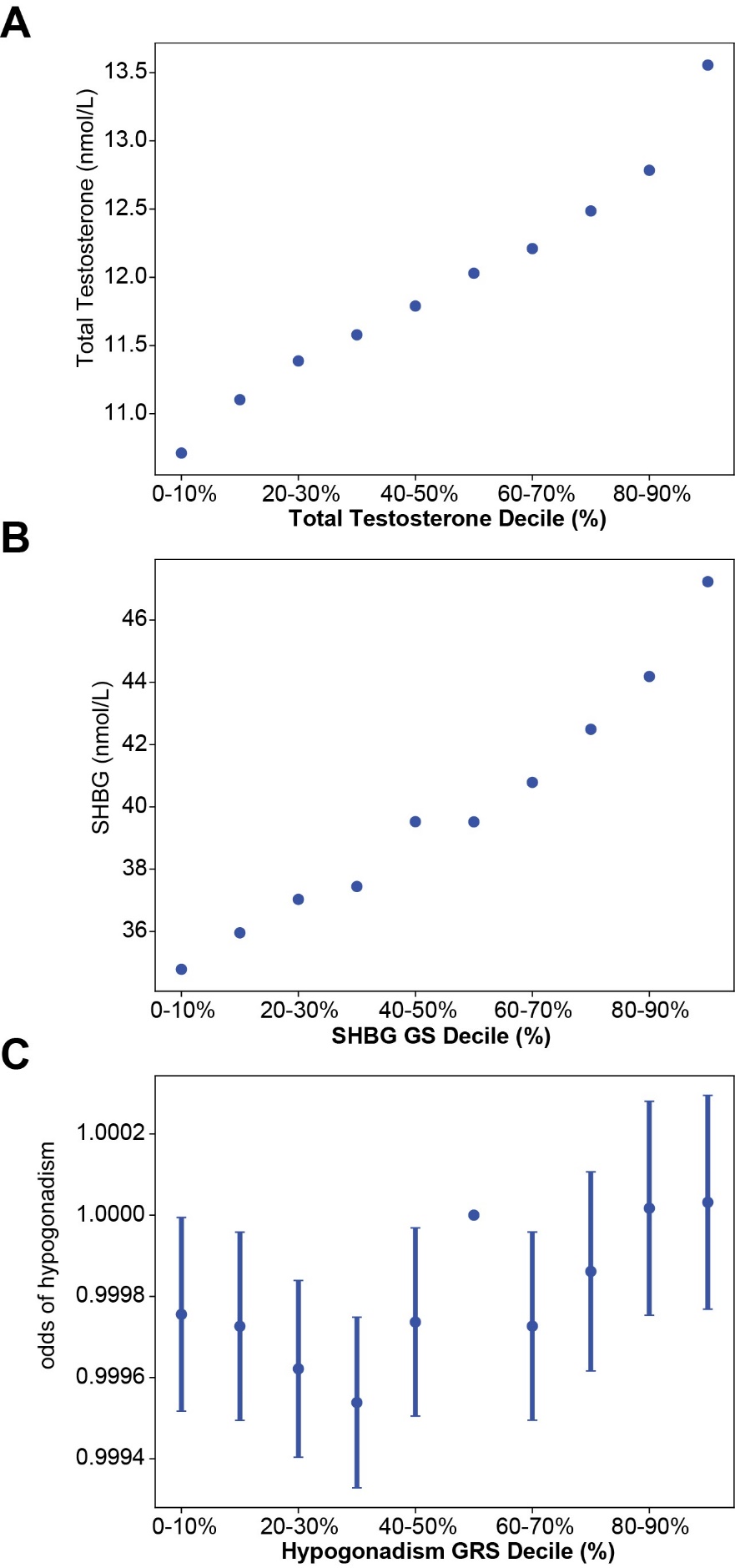


**Figure S9: MR of Total Testosterone, SHBG and Hypogonadism and Disease Incidence in MVP EUR. (A)** Plot of MR effect sizes (beta) for MVP EUR total testosterone**,** SHBG, and hypogonadism and gout, type 2 diabetes, obesity, hyperlipidemia, and NAFLD. MR scatter plots of total testosterone**,** SHBG, and hypogonadism MVP EUR effects and NAFLD **(B),** type 2 diabetes **(C)**, gout **(D),** hyperlipidemia **(E),** obesity **(F)**, chronic heart disease **(G).**

**
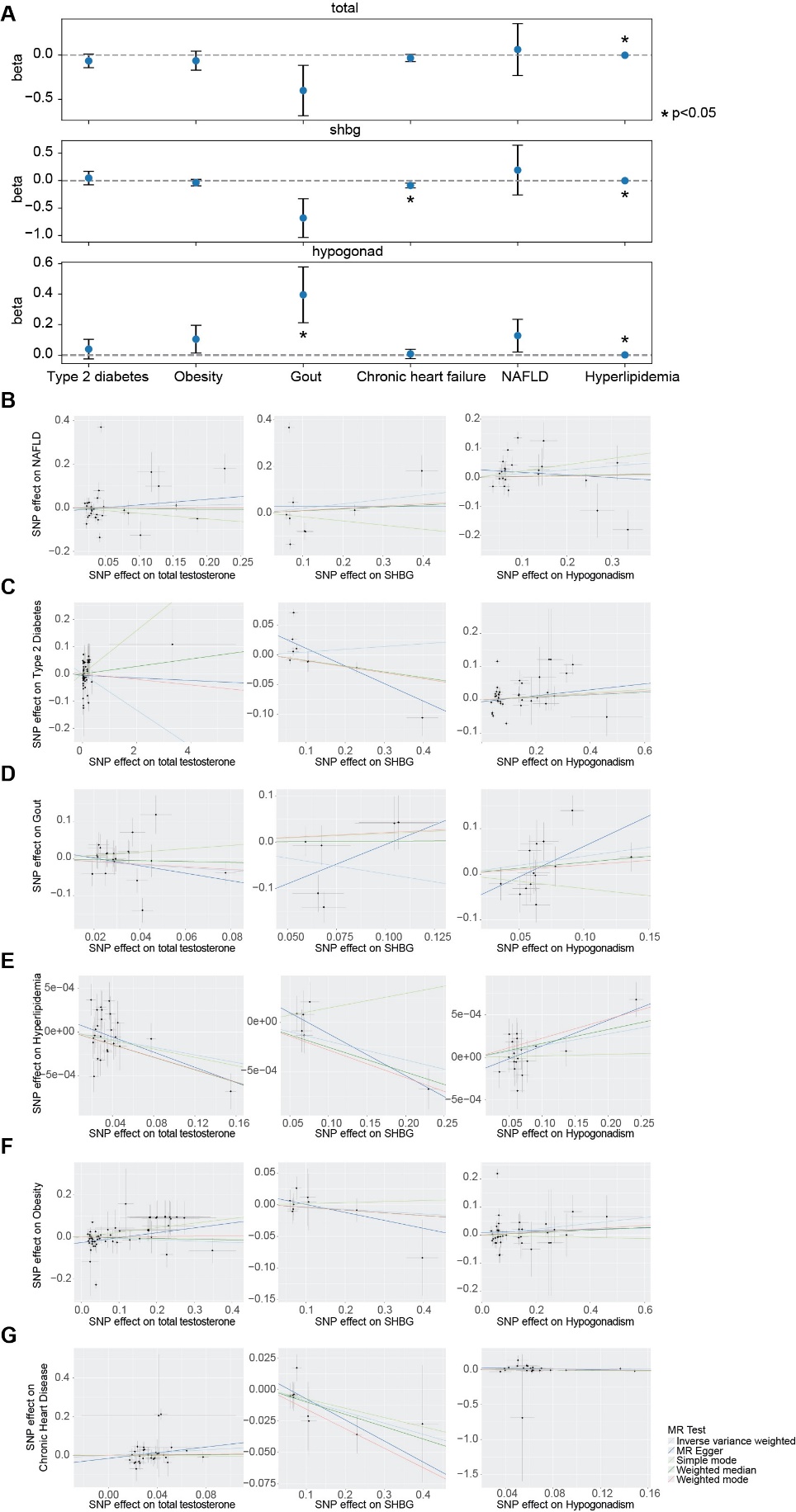
**

**Figure S10: Association of Total Testosterone, SHBG GS and Hypogonadism GRS with Liver Disease.** CoxPH Hazards ratio of total testosterone GS, SHBG GS and hypogonadism GRS and phecode 571 - chronic liver disease **(A),** phecode 572 - ascites (**B),** and phecode 573 - liver necrosis **(B)** in EUR, AFR, AMR, EAS ancestry groups in MVP.


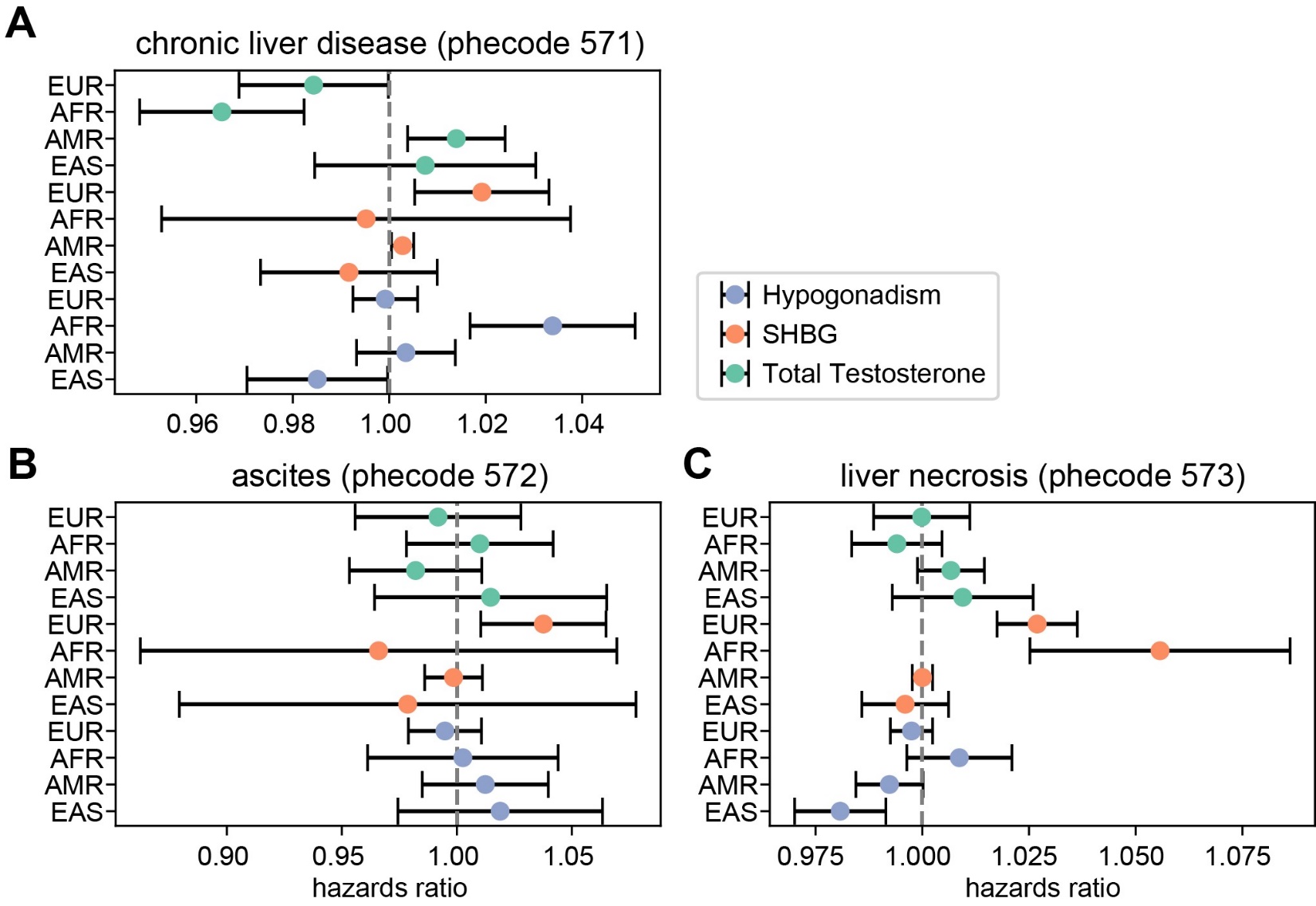


**Figure S11: Association of Total Testosterone, SHBG GS and Hypogonadism GRS with Testicular Dysfunction, Prostate Cancer, Dementia Risk.** CoxPH Hazards ratio of total testosterone GS, SHBG GS and hypogonadism GRS and phecode 257 - testicular dysfunction **(A),** phecode 185 - prostate cancer (**B),** and phecode 290 - dementia **(B)** in EUR, AFR, AMR, EAS ancestry groups in MVP. Asterisk (*) indicates associations significant after Benjamini-Hochberg multiple test correction. Full association statistics are given in Supplementary Table 10.


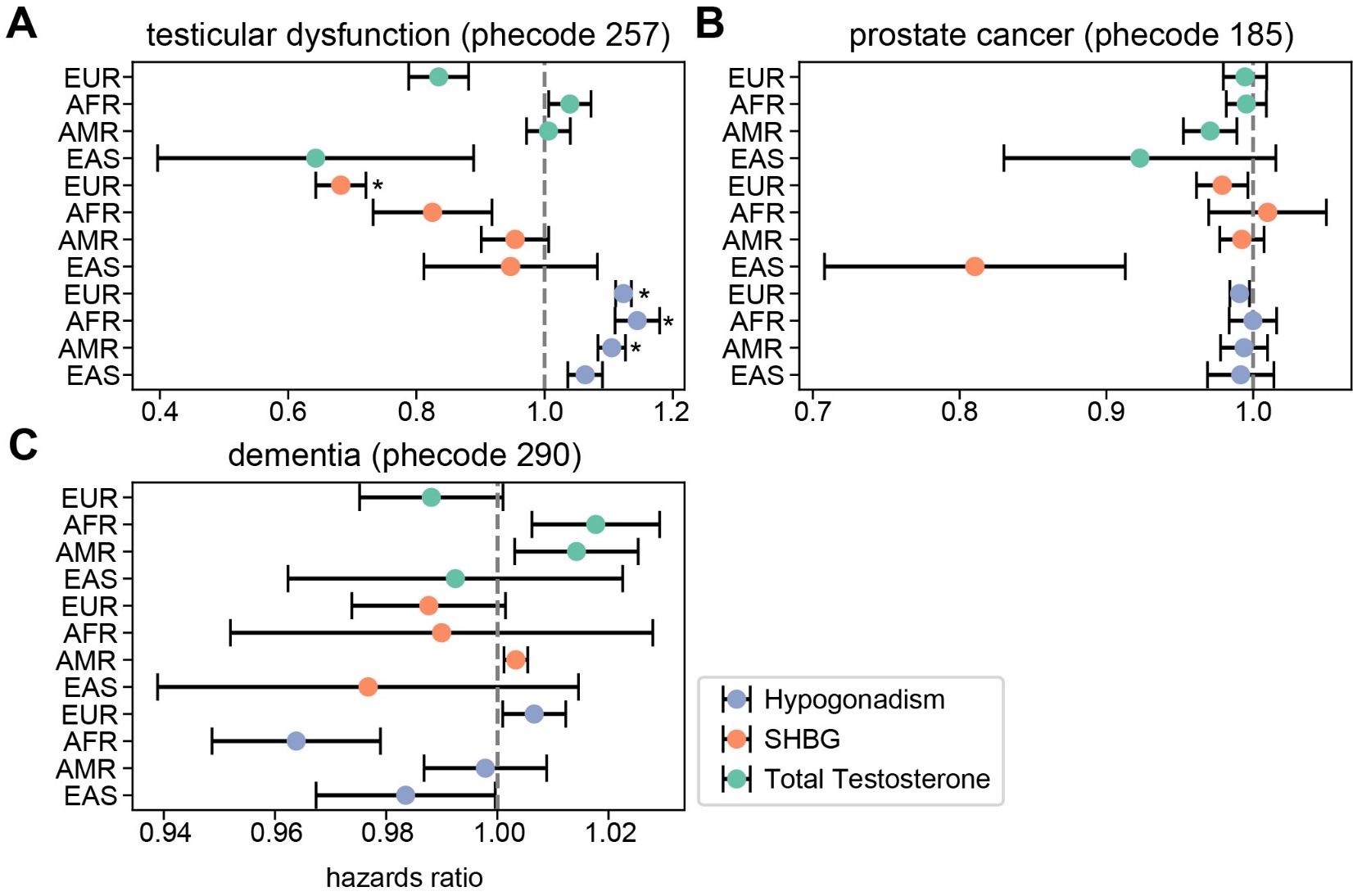

Supplement: Supplementary file 1 — Supplemental Information [file 41467_2025_57372_MOESM1_ESM.docx]
