# Supplementary material for: Discovery of novel ancestry specific genes for androgens and hypogonadism in Million Veteran Program Men
Source: Nat Commun. 2025 May 2;16:4104. doi: 10.1038/s41467-025-57372-x (PMC12048691; doi:10.1038/s41467-025-57372-x)
Supplement: Supplementary file 2 — Description of Additional Supplementary Files [file 41467_2025_57372_MOESM2_ESM.pdf]

Supplementary Datasets

File Name: Supplementary_dataset_1
Description: MVP GWAS Results for Total Testosterone, Free Testosterone, SHBG levels and Hypogonadism in EUR, AFR, AMR and EAS Men

File Name: Supplementary_dataset_2
Description: MVP GWAS Variants Not Validated in UK Biobank

File Name: Supplementary_dataset_3
Description: GTEx Gene Expression Colocalization Results

File Name: Supplementary_dataset_4
Description: MVP Ancestry Group PheWAS Colocalization Results

File Name: Supplementary_dataset_5
Description: MVP Meta-Analysis Results for Total Testosterone, Free Testosterone, SHBG levels and Hypogonadism

File Name: Supplementary_dataset_6
Description: Novel Meta-Analysis Variants Identified in MVP Analyses MVP

File Name: Supplementary_dataset_7
Description: Details of Publicly-Available GWAS Used for MR Studies of Total Testosterone, SHBG levels and Hypogonadism Risk

File Name: Supplementary_dataset_8
Description: Cox Proportional Hazards Analysis Results with Disease Risk
